# Standard of Care and Promising New Agents for Triple Negative Metastatic Breast Cancer

**DOI:** 10.3390/cancers6042187

**Published:** 2014-10-24

**Authors:** Patrizia Mancini, Antonio Angeloni, Emanuela Risi, Errico Orsi, Silvia Mezi

**Affiliations:** 1 Department of Experimental Medicine, Sapienza University of Rome, Viale Regina Elena 324, Rome 00161, Italy; 2 Department of Molecular Medicine, Sapienza University of Rome, Viale Regina Elena 324, Rome 00161, Italy; E-Mail: antonio.angeloni@uniroma1.it; 3 Department of Radiology, Oncology and Human Pathology, Sapienza University of Rome, Viale Regina Elena 324, Rome 00161, Italy; E-Mails: emanuela.risi@libero.it (E.R.); silvia.mezi@uniroma1.it (S.M.); 4 Department of Surgical Science, Sapienza University of Rome, Viale Regina Elena 324, Rome 00161, Italy; E-Mail: errico.orsi@uniroma1.it

**Keywords:** TNBC, VEGF, Aurora, Hsp90, PARP, immunotherapy, platinum salts, EGFR, PI3K/AKT, androgen receptor

## Abstract

Triple negative breast cancer (TNBC) is a cluster of heterogeneous diseases, all of them sharing the lack of expression of estrogen and progesterone receptors and HER2 protein. They are characterized by different biological, molecular and clinical features, including a poor prognosis despite the increased sensitivity to the current cytotoxic therapies. Several studies have identified important molecular features which enable further subdivision of this type of tumor. We are drawing from genomics, transcription and translation analysis at different levels, to improve our knowledge of the molecular alterations along the pathways which are activated during carcinogenesis and tumor progression. How this information should be used for the rational selection of therapy is an ongoing challenge and the subject of numerous research studies in progress. Currently, the vascular endothelial growth factor (VEGF), poly (ADP-ribose) polymerase (PARP), HSP90 and Aurora inhibitors are most used as targeting agents in metastatic setting clinical trials. In this paper we will review the current knowledge about the genetic subtypes of TNBC and their different responses to conventional therapeutic strategies, as well as to some new promising molecular target agents, aimed to achieve more tailored therapies.

## 1. Introduction

Triple negative breast cancer (TNBC) represents a cluster of heterogeneous diseases showing distinct molecular, pathological and clinical features. Phenotypically, TNBC is characterized by the lack of estrogen (ER) and progesterone (PR) receptors expression, as well as that of human epidermal growth factor receptor 2 (HER2) gene amplification/overexpression [1]. Moreover, the same characteristics are more frequently observed in TNBC compared with other BC subtypes as the upregulation of cytokeratins 5, 14, 17 and elevation of the epidermal growth factor receptor 1 (EGFR1) [2,3,4]. They are frequently associated with high expression of proliferation markers, *i.e.*, Ki67 [3], high levels of cyclin E, low levels of cyclin D1 [5,6] and activation of the beta-catenin pathway [7]. In addition, more than 50% of TNBC show P53 nuclear expression [8]. The phenotypic classification based on immunohistochemical (IHC) analysis of ER, PR and HER2 is typically used in clinical practice for decision-making to identify TNBC. Intrinsic molecular breast cancer (BC) whose classification is based on the analysis of global gene expression identifies four main molecular subtypes of BC: luminal A, luminal B, HER2, basal-like, and the more recently identified claudin-low tumor subtype [9,10] Most of the basal-like subtypes lack the expression of ER, PR and HER2, so they are frequently identified as TNBC. However, there is a discordance rate of 20%–30% between basal-like and TNBC [11,12,13,14,15].

This data came from three large clinical trials (GEICAM/9906 [16], MA.12 [17] and MA.5 [18]), in which TNBC is subtyped using the PAM50 qRT-PCR-based assay. This incomplete overlap between TNBC and basal-like subtypes suggests that TNBC is a heterogeneous group of tumors for which a deeper subclassification is needed [19]. TNBC occurs in about 10%–20% of invasive breast cancers, more frequently affecting premenopausal women and are more prevalent in the African-American population [20,21]. Tumors with a triple negative (TN) phenotype are characterized by poor clinical prognostic features; they are usually larger in size, higher in grade, with earlier lymph node involvement, turning into aggressive tumor behaviour and worst outcomes for patients [1]. Therefore, only a few TNBC patients present a good prognosis. For example, adenoid cystic and medullary carcinoma are predominantly negative for ER, PR, and HER2, but, despite the high proliferation index, they have been consistently reported to have excellent prognoses [22]. Another condition for a better prognosis may correlate with the presence of high levels of tumor infiltrating lymphocytes [23]. The incidence of BRCA mutations in TNBC varies from 16% to 42% [24,25]. This mechanism of BRCA1/2 downregulation, that occurs by epigenetic alterations and overexpression of BRCA inhibitors, contributes to the aneuploidy and genomic instability which is frequent in TN disease [26,27]. A recognized factor linked to TNBC pathogenesis is the metabolic syndrome, which consists of central obesity, insulin resistance, impaired glucose tolerance, dyslipidemia and hypertension [28]. In particular, Davis has reviewed the role played by the insulin-leptin-adiponectin axis in TNBC tumorigenesis and progression [28]. Particularly, the intracellular control of the survival/apoptosis balance, as well as proliferation, cell-cycling, angiogenesis is impaired by heightening leptin and reducing adiponectin levels [29]. Insulin may mediate BC risk via both direct and indirect effects, resulting in increased concentration of androgens and estrogens, along with increased concentration of IGF-I [30,31,32,33,34]. Although TNBC is a minority among all types of BC, they develop into a full blown metastatic disease and play a significant part in the mortality rate for BC because their poor prognosis and high risk of recurrence [35,36,37]. Metastatic TNBC is an aggressive disease that is associated with visceral and central nervous system metastases [38]. The 5-year survival of patients with TNBC is less than 30%, and almost all of them will die because of the progression of their disease despite adjuvant chemotherapy [39]. Current systemic treatment options for metastatic TNBC patients are primarily represented by cytotoxic chemotherapy. Although chemotherapy remains the mainstay of treatment for metastatic TNBC, there are no standard chemotherapic schedules to date. However, trials have been conducted predominantly in populations of unselected patients.

This review explores the biological features and clinical behaviour of TNBC according to tumor heterogeneity and gene expression profiles (GP). Our aim is to revise the indications to specific therapeutic strategies, which would have to be different for each specific TNBC subtype, according to the GP. Indeed, advances in molecular characterization and classification of the disease into subtypes could really change the treatment options allowing a rational choice for targeted therapies. Finally, in order to optimize the treatment of metastatic TNBC, we propose that the future clinical trials should be focused on the patient’s biological differences as they emerge from gene expression profiles.

## 2. Genetic Subtyping of TNBC: Differential Sensitivity to Chemotherapy According to Gene Prophile, Current Treatment and Novel Strategies

The biological heterogeneity enables a molecular stratification of TNBC to be made. Each subtype, identified by its specific molecular characteristics, could be treated with specific agents, targeted on the molecular biomarker profiling. The BC intrinsic subtypes may reflect some stop at different stages in the normal development of epithelial cells. Perou stratifies TNBC in HER2-enriched, basal-like, and claudin-low subtype [11]. The claudin-low group shows morphological features similar to mammary stem cells, from which it takes its origin. Moreover, this group is characterized by a high enrichment for markers of epithelial to mesenchymal transition (EMT), *i.e.*, the changing from the epithelial morphology to a mesenchymal phenotype, the expression of genes of the immune response and the low or absent expression of the luminal differentiation marker. Therefore, in these patients it would be more appropriate to consider a treatment that affects the cancer stem cells. The next step on the path of differentiation is the bipotential luminal progenitor (luminal and myoepithelial) that gives rise to a basal-like phenotype. The basal-like phenotype represents the majority of TNBC and is discussed later. HER2-enriched subtype arises from cells in a further phase of development. This subtype takes its origin from the transformation of a progenitor which is characterized by a loss of basal characteristics and gains characteristics of luminal: a late luminal/basal cell. The HER2-enriched triple negative tumours are not HER2+ or HER2 amplified, as if they were, for example, carriers of a mutation of HER2 in the kinase domain. Activity of HER2-targeted agents in patients with HER2-enriched subtype-that are clinically HER2-negative, is unknown because, unfortunately, they are not included in studies focusing on the activity of anti HER2 agents.

Based on DNA microarray expression profiling, Lehmann and colleagues [40,41] reported that TNBC could be classified into seven subtypes, labeled as basal-like 1 (BL1), basal-like 2 (BL2), immunomodulatory (IM), mesenchymal (M), mesenchymal stem-like (MSL), luminal androgen receptor (LAR) and unstable (UNS).

Basal-like classes, BL1 and BL2, represent the main components of TNBC with a rough frequency of 80%. They are characterized by the enrichment of genes that regulate cell cycle, by cell-cycle checkpoint loss, and by elevated DNA damage response pathways. In 10% to 20% of TNBC is possible to find a BRCA mutation; indeed, most BRCA1 mutated BC fall into the BL1 and BL2 subtypes. Lehmann’s analysis confirmed most of TNBC resulting to be classified as basal-like molecular subtype (49%), being more rare the other subtypes (14% luminal A, 11% normal breast-like, 8% luminal B, 5% HER2, 13% unclassifiable).

The UNS, BL1, BL2, and M subtypes expressed higher levels of basal cytokeratin, differently from tumors within the LAR subtype expressing high levels of luminal cytokeratins and other luminal markers. The stromal and immune gene clusters, which identify gene expression patterns coming from the microenvironment, are critical for the identification of Lehmann’s M, MSL, and IM subtypes, respectively [42].

The seven subtypes classification has been revalidated by Masuda and collegues, who investigated the clinical relevance of TNBC heterogeneity [39]. Their analysis highlights that TNBC subtypes have different pathological complete response rates (pCR) to standard neoadjuvant chemotherapy with sequential taxane and anthracycline-based regimens. The authors’ conclusion was that TNBC classification by seven subtypes was able to predicts high *versus* low pCR rate. These data strongly suggest that the classification in subtypes may encourage innovative strategies for personalized medicine in patients with TNBC (Table 1).

### 2.1. BL1 Subtype

Lehmann reported that the BL1 subtype shows a high level expression of genes involved in cell cycle division and DNA damage response, suggesting that this tumor subtype would preferentially respond to antimitotic agents, such as taxanes [40]. Indeed, TNBC patients, whose tumors correlated to the basal-like subtype, had a significantly higher pCR (63%; *p* = 0.042) when treated with taxane-based therapies compared to mesenchymal-like (31%) or LAR (14%) subtypes [42].

In addition, these data were confirmed by Masuda [39], who observed that BL1 tumors are the most chemosensitive and have the greatest number of pCR rate (52%) with standard, taxane based, neoadjuvant regimens when compared to other subtypes. In metastatic setting, several trials suggest a lack of specific benefits from taxanes for TNBC against other subtypes of BC and, generally, they support the conclusion that taxanes are effective in all subtypes of BC, despite the overall survival (OS) was significantly worse for the TNBC compared to hormone receptor positive disease [43].

**Table 1:** Clinical relevance of the heterogeneity in TNBC. The classification into subtypes can differentiate diseases by their gene expression profiles and relative chemosensitivity, encouraging innovative approaches to personalized therapies. The gene expression profiling of primary TNBC allows to stratify them in different subtypes. The stratification into subtypes has clinical value and is able to differentiate each primary disease in terms of chemosensitivity. For each subtype on the basis of its molecular profile, the best tailored treatment has been proposed.

	Gene Expression	Therapeutic Agents	pCR to CT	CT
BL1	Cell cycle pathway, cell division pathway	Taxanes/Anthracicline Platinum	52%	+++
	Proliferation pathway (AURKA, AURKB, MYC, NRAS)	Aurora kinases inhibitor (AMG900, AS703569)		
	DNA damage pathway (ATR/BRCA pathway)	PARP-inhibitor (iniparib, olaparib, veliparib)		
	RNA Polymerase			
BL2	EGF pathway	Cetuximab, erlotinib, gefitinib	0	−
	IGFIR pathway	BMS-754807		
	MET pathway			
	NGF pathway			
	WNT/B-catenin pathway			
	Glicolisis/Gluconeogenesis			
IM		Taxanes/Anthracicline	30%	+
	TH1/TH2 pathway, T cell receptor signaling	Platinum/Lambrolizumab (MK-3475)/Nivolumab		
	Cytokine signaling	PLX3397 (CSF-1 inhibitor)		
	DC pathway, NK cell pathway	Indoximod (IDO inhibitor )		
	B cell receptor signaling pathway			
	NFκB, TNF, JAK/STAT signaling			
	CTL4, IL12, IL7 pathway	Ipilimumab		
	Antigen processing/presentation			
	DNA damage pathway (ATR/BRCA pathway)	Platinum		
M		Taxanes/Anthracicline	31%	+
	Cell motility pathway (Regulation of Actin by RHO)	Dasatinib		
	Cell differentiation pathway (WNT/B-catenin, ALK, TGFβ)	Windorphen		
	IGF/mTOR pathway	NVP-BEZ235		
	ECM receptor interaction pathway			
MSL		Taxanes/Anthracicline	23%	+
	Cell motility pathway (Regulation of Actin by RHO, RAC1) EMT-associated genes/low expression of claudin low 3,4,7 Smooth muscle contraction	Dasatinib		
	Cell differentiation pathway (WNT/B-catenin, ALK, TGFβ)	Windorphen		
	Angiogenesis-associated genes	Bevacizumab		
	Growth factor signaling pathway (EGF, PDGF, calcium signaling, GPCR, ERK1/2, ABC transporter, adipocytokine signaling, PI3K-AKT-mTOR pathway)	NVP-BEZ235		
	ECM receptor interaction pathway			
	T cell receptor signaling/NK cell pathway/NFκB signaling			
LAR		Taxanes	10%	+/−
	Hormonally regulated pathways (steroid synthesis, porphiryn metabolism, androgen/estrogen metabolism)	Abiraterone/bicalutamide/enzalutamide		
	PI3K/mTOR/AKT pathway; HSP90	NVP-BEZ235; 17-DMAG (HSP90 inhibitor)		

However, in these trials, TNBC was unselected based on gene ontologies and differential gene expression profiles. In the randomized open-label phase III CALGB 40502/NCCTG N063H, the new and expensive BC treatments, nanoparticle albumin bound nab-paclitaxel (Abraxane) and ixabepilone, the latter being a potent epothilone that can be effective after microtubule inhibitor resistance (Ixempra), have failed to demonstrate any superior efficacy *versus* the standard of weekly paclitaxel in combination with bevacizumab in patients with chemotherapy naïve metastatic BC. Moreover, weekly paclitaxel showed a better toxicity profile [44]. Even in these study TNBC was unselected, and subtypes ignored. The effectiveness of treatment reported is evalued as average effect between the different subtypes. Indeed, BRCA1 mutation might confer decreased response to docetaxel in comparison with sporadic forms of TNBC [45,46]. The role of docetaxel and carboplatin for the treatment of metastatic TNBC with BRCA-mutation will be explained in the TNT trial NCT00532727. An additional therapeutic strategy may be based on the pathways of elevated DNA damage response (ATR/BRCA). As BRCA1/2 are critical regulators of DNA repair and maintenance of genomic stability [47], it was supposed that TNBC may be particularly sensitive to agents that cause DNA damage, including platinum-containing compounds that induce lethality in repair-defective cells via inhibition of poly(ADP-ribose) polymerase (PARP)1/2 pathways [48]. Preclinical data confirmed that platinum agents may be particularly active in TNBC and BRCA1 associated BC. Several authors have showed *in vitro* an increased sensitivity to the DNA cross-linking agents in BRCA1 deficient cells. Interestingly, this specific sensitivity can be reversed restoring BRCA1 function or inducing its upregulation [49,50,51,52].

In fact, high prevalence of BRCA1 dysfunction was identified in basal-like BC [27]. Although clinical data are controversial, high responsiveness to cisplatin have been seen in patients with triple-negative and BRCA1-associated BC, with pCR rates of over 80%, while pCR rates with cisplatin for sporadic TNBC were considerably lower, around 20% [53,54]. The expected response rate (RR) to platinum agents in unselected patients with BC ranges from 10% (pretreated) to 25% (chemotherapy naive) [55]. Although TNBC may be more chemosensitive in general [56], nevertheless RR to cisplatin in first- and second-line treatment of TNBC was only 10% in a recently reported trial [57]. Outcomes for platinum-containing agents administered as monotherapy for metastatic TNBC have been poor [58]. Indeed, platinum doublet or triplet therapy appears more active [59,60,61]. A recent meta-analysis about the role of platinum-based chemotherapy in TNBC, demonstrated that during neo-adjuvant chemotherapy the clinical complete response (cCR) and the pCR rates were significantly higher for the TNBC group treated with platinum based chemotherapy compared to the non-TNBC group [62]. However, in advanced/metastatic BC, the cCR, partial response (PR) and the disease control rates for the TNBC group were not significantly different compared to the non-TNBC group. The 6-month progression-free survival (PFS) rate for the TNBC group was higher than that one of the non-TNBC group in all patients, although the 1- and 2-year PFS rates were not significantly different. Furthermore, the PFS rates were not significantly different between groups in patients with advanced/metastatic BC. The meta-analysis conclusions were that platinum-based chemotherapy in BC patients with TNBC showed an improved short-term efficacy compared to the non-TNBC group during neo-adjuvant chemotherapy, but has not yet been demonstrated to have an improved effect in advanced BC. Unfortunately, the systematic meta-analysis included studies where the overall quality was not high, and would have to require more rigorous design of high-quality randomized controlled studies in order to determine definitively the role of the platinum salts in TNBC. Platinum based-therapies were widely discussed during ASCO 2014. An important phase II study including only metastatic TNBC patients (TBCRC009) was presented by Isakoff [63]. The study aimed to identify biomarkers predicting response to single-agent platinum chemotherapy (cisplatin or carboplatin) administered as first or second line. None of the established biomarkers (p63/p73 expression, p53/PIK3CA mutation, molecular subtype) was found to predict response. Only Homologous Recombination Deficiency (HRD) assay associated with defects in the BRCA 1/2 pathway may reveal a positive correlation with platinum sensitivity and subsequently may be considered a response predictor. Other conclusions of the study were that platinum monotherapy is active in metastatic TNBC (ORR 25.6%) and that BRCA1/2 carriers were not associated with longer PFS or OS compared to BRCA1/2 WT.

The existing clinical data do not support preferential use of platinum agents in sporadic TNBC in metastatic setting, to date. In fact, the current data are insufficient to recommend the use of platinum salt as the standard treatment in earlier lines, although its use is not contraindicated in metastatic setting. This might change following the results of ongoing studies: the large randomized phase III TNBC Trial (NCT00532727) will eventually help to define how platinum should be utilized in TNBC metastatic disease.

Through a strategy of selection, a molecular pathway by which cisplatin induces cell death in TNBC has been discovered [64]. The p63/p73 network mediates chemosensitivity to cisplatin in a biologically defined subset of primary TNBC, suggesting that these triple-negative cancers may share the highest cisplatin sensitivity of BRCA1-associated tumors. The possibility of selecting a subgroup of triple negative platinum responsive tumors, as well as the ongoing analyses of additional markers for platinum sensitivity, are both particularly exciting, so that the results of prospective studies are eagerly awaited. Moreover, in advanced TNBC, platinum agents have been combined with targeted agents such as bevacizumab [65], cetuximab [57,66], erlotinib [67] and iniparib [68]. A randomised phase II trial demonstrated promising overall response rates (ORR) when carboplatin was added to single-agent cetuximab in pretreated advanced TNBC patients [69], although the data from PFS and OS is pending. BRCA1 and BRCA2 have a pivotal role in DNA double-strand break repair [70]; this makes BRCA-mutant to be sensitive to some DNA-damaging agents such as cisplatin [71], as well as to PARP inhibitors [72]. Indeed, PARP is one of the most interesting and promising target for treating TNBC. PARPs are a family of enzymes which are involved in many cellular functions, including DNA repair, genomic stability, cell cycle progression and apoptosis [73]. Among different members of the family, PARP-1 and PARP-2 are the most common enzymes which are activated by DNA damage. These enzymes show an overlapping functional activity and similar substrate molecules, although PARP-1 accounts for about 80% of total cellular PARP function in mammalian cells. Both PARP-1 and PARP-2 work as DNA single strand break (SSB) sensors [70]. As one strand of DNA breaks, PARP-1/2 bind to the damaged site, so activating the cascade of events which lead to DNA repair. PARP-1/2 are essential in the repair of SSB, in fact, deficiency in the PARP-1/2 activity leads to the accumulation of SSB [70]. Since, both BRCA1 and BRCA2 are essential components of the homologous recombination (HR) complex, inhibition of PARP-1/2 in BRCA1 and BRCA2-deficient cancer cells, results in synthetic lethality, due to the improper repair of the endogenous daily DNA damage [74]. As a result, specific inhibitors of PARP-1/2 hit cancer cells with two mutated copies of these genes, whilst saving normal cells with almost one right copy of them, leading to an eventual reduction in toxicity. Several PARP-1 inhibitors have undergone clinical trials. Olaparib is an oral active small molecule PARP-inhibitor. As the phase I and II trials have been completed [75], a phase III randomized controlled multi-centre trial was designed to assess the efficacy and safety of the drug in metastatic BC patients with BRCA1/2 mutations (NCT02000622); however it is not yet open for participant recruitment. Iniparib has been showed to be active in combination with chemotherapy in a phase II trial. The addition of iniparib improved PFS and OS (12.3 months *versus* 7.7 months), with no significant increase in toxicity [68]. Despite encouraging data from phase II trials, iniparib has not confirmed its efficacy in the phase III trial [76], a possible explanation being the heterogenous nature of TNBC; indeed, an alteration of the DNA-repair pathway is needed for PARP-inhibitor activity. Therefore, improved selection of patients is expected to give better results for iniparib, even if the mechanism of its antitumoral activity is not fully understood and it is no more considered as part of PARP-inhibitor class. Another PARP inhibitor, veliparib, was tested as single agent in patients with refractory tumors and lymphomas. It has also been shown to be active in combination with carboplatin [77,78]. Veliparib was administered in combination with temozolamide in BC patients. A phase II trial including 15 TN and eight BRCA mutated patients, showed that PFS was 5.5 months in the BRCA-mutated subgroup *versus* 1.8 months for non mutated patients, suggesting a possible role of veliparib in BRCA mutated BC [79]. In another phase II study (I-SPY 2), 134 HER2-negative patients were randomized to receive carboplatin plus veliparib prior to a neoadjuvant chemotherapy regimen that included carboplatin. Addition of carboplatin/veliparib doubled the pCR rate from 26% to 52% [80]. Unfortunately, the study design does not allow the relative contributions of veliparib and carboplatin to be determined. During ASCO 2014 a phase II trial was presented in which veliparib was administered as a single agent followed by post-progression therapy of veliparib plus carboplatin in patients with BRCA-associated metastatic BC. Veliparib was proved to be active in BRCA1/2 BC with an RR in the same range as the other PARP inhibitors. A poor response was observed after crossover to carboplatin and veliparib. This probably means that the best strategy could be the combination of platinum and veliparib followed by veliparib alone [81].

Promising results were also reported for BMN 673, which is a PARP inhibitor in clinical evaluation in BC patients with deleterious germline BRCA 1 and 2 mutations. Of the 18 patients treated with BMN 673, eight had partial responses [82].

Another promising class of agents in BL1 tumor subtype is the Aurora kinases that are a family of serine/threonine kinases which play a crucial role in the shaping of mitotic spindles by regulating chromosome alignment and segregation, centrosome duplication and cytokinesis [83,84]. There are three human homologues members: Aurora A, B and C, which share similar *C*-terminal domains, but have different *N*-terminal ends. Aurora C, the less extensively studied member of the Aurora family, is present only in mammals, predominantly expressed in testis [85], and it has been shown to have a possible functional overlap with Aurora B in meiosis [86,87]. Aurora A and B kinases are expressed in most normal cells, but they have different cellular localization and functions during mitosis [83] interacting with a distinct set of proteins. The best studied Aurora A substrate is TPX2, which induces activation of the protein by auto-phosphorylation of Thr288 [88,89]. Other substrates include Ajuba, Eg5 and CDC25B [85]. In addition to its role in mitosis, Aurora A also regulates the functions of p53 and NF-κB. Moreover, Aurora A interacts with BRCA1, colocalizes with BRCA1 on centrosome and activates it by phosphorylation of serine 308 [83]. Aurora A is overexpressed or amplified in several human epithelial tumors, including breast, lung, prostate, colon, ovary and pancreatic cancers. In particular, TNBC Aurora A was shown to be overexpressed at both RNA and protein levels, and it seems to be associated with a poor prognosis [90]. Aurora B is a chromosome passenger protein, associated with the chromosomal passenger complex (CPC), which comprises the inner centromere protein INCENP, and the targeting proteins survivin and borealin [83]. Aurora B localizes to the kinetochores from prophase to metaphase and relocates to the central spindle and the midbody in cytokinesis [91] performing three distinct functions: it is a histone kinase involved in phosphorylation of chromatin proteins, *i.e.*, histone H3, a spindle checkpoint kinase and a cytokinesis kinase [85]. Mitotic centromere-associated kinesin (MCAK) is an Aurora B substrate *in vitro* and *in vivo* and is required for chromosome gathering, biorientation, bipolar spindle formation and chromosome movement during anaphase [92]. MCAK is inactivated by Aurora B [92] and activated by different proteins, such as cdc14 or the inactivation of Aurora B, suggesting that MCAK activity is modulated by phosphorylation and dephosphorylation during the cell cycle. Given the importance of MCAK in Aurora B activity, it could be a good target for Aurora B inhibition.

The involvement of Aurora A and B kinases in carcinogenesis has made these proteins a good target for cancer therapy, so that several inhibitory drugs have been developed against Aurora A and/or Aurora B. Targeting Aurora kinases could represent a new effective approach for TNBC treatment. TNBC cell lines were found to be more sensitive to the pan-inhibitor of Aurora kinases, AS 703 569 when compared to other types of BC cells. Inhibition of proliferation was associated with cell-cycle arrest, aneuploidy, and apoptosis. In addition, targeting Aurora kinases by a single agent or in some chemotherapic combination significantly inhibited tumor recurrence *in vivo* [93]. Moreover, overexpression of Aurora A predicted poor OS and PFS in TNBC [90]. A phase II study of the Aurora and angiogenic kinase inhibitor ENMD-2076 in previously treated locally advanced and metastatic TNBC is currently ongoing (NCT01639248).

### 2.2. BL2 Subtype

Lehmann reported that BL2 subtype only moderately correlates to the basal-like molecular class (31%), with a portion of tumors unclassified (22%) and features of basal/myoepithelial origin, as demonstrated by higher expression levels of *TP63* and *MME* (CD10) [40,41].

According to their genetic profile, that involves growth factor signaling, BL2 subtype is chemoresistant, as evidenced by the lower rate-pCR compared to other subtypes of TNBC. None of them has achieved a pCR, as reported by Masuda [39]. BL2 displays unique gene ontologies involving growth factors signaling. Several types of cancer, including BC, carry deregulation of EGFR-mediated signaling by different molecular mechanisms, such as overexpression, acquisition of activating mutations of the receptor and activation induced by ligands, which act in autocrine/paracrine manner [94]. However, several clinical studies reported that targeting HER1 in BC yielded no credible results [95,96]. Two classes of EGFR inhibitors are currently available in clinical: monoclonal antibodies directed against the receptor, such as cetuximab and panitumumab, and the small-molecule tyrosine kinase inhibitors (TKIs) gefitinib and erlotinib. Monoclonal antibodies anti-EGFR are designed specifically to bind the receptor with higher affinity than either EGF or TGF-α, thus blocking phosphorylation of EGFR. Agents targeting a single member of the HER family inhibit signaling through competitive, reversible binding to the EGFR/HER1 tyrosine kinase domain. Conversely, the pan-HER inhibitor binds irreversibly (*i.e.*, by a covalent bond) to the adenosine triphosphate domain of each kinase-active members of the HER family. Cetuximab and panitumumab are approved for the treatment of advanced colorectal cancers (CRC) lacking *pan RAS and B raf* mutations [97], while gefitinib and erlotinib may be used for treating advanced non-small cell lung cancers (NSCLC) that express mutant forms of *EGFR* [97]. Despite that, the sensitivity to cetuximab in basal/TN cell lines seems to be poor [98]. The addition of cetuximab to irinotecan and carboplatin in first- and second-line MBC patients in the USOR-04-070 trial [99], resulted in improved response rates in a subset of TNBC patients. However, no improvements in either PFS or OS were emerging for the TNBC subgroup, and the cetuximab combination resulted in a substantial increase in diarrhea compared to chemotherapy alone.

The randomized phase II EGFR trial, BALI-1, prospectively evaluated the addition of cetuximab to cisplatin for the treatment of first- and second-line TNBC patients (*n* = 173) [68]. The combination was safe, with minimal increased acne type skin rashes, but the combination arm failed to improve ORR or OS. A significantly increased median PFS to cisplatin alone was reported, but PFS gains may have been due to the inferior performance of the non-standard control arm. Another cetuximab trial, which added carboplatin to cetuximab, was performed in heavily pretreated TNBC patients (*n* = 102). Patients were randomized to receive the cetuximab alone or in combination with carboplatin, added after progression or as concomitant therapy from the beginning. Preliminary results have shown an ORR of 17%, and prolonged PFS was seen in responders compared to the overall trial population [69]. The final results of the trial were reported by Carey in 2012 [100]. On the cetuximab plus carboplatin arm of 71 mTNBC patients, the ORR was 17%, with 1 having experienced complete and 11 partial responses. Furthermore, 10 had stable disease SD prolonged. Interestingly, the authors reported long-term responses of 1 year in two patients treated with cetuximab monotherapy and two in the combination therapy arm. The same authors also assessed the predictive value of the signature of EGFR expression before and after 7–14 days of treatment with cetuximab. A significant clinical benefit was seen mainly in cases characterized by a high basal expression of EGFR and low expression of the same signatures after 7–14 days of treatment, regrettably, EGFR pathway inhibition was apparent in only a minority of tumors 7 to 14 days after beginning anti-EGFR therapy, demostrating that most TNBC tumor samples had activation of the EGFR pathway, but only a minority showed pathway inhibition with cetuximab. This data suggests that either cetuximab is ineffective against this target, or that in TNBC there are alternative mechanisms that do not depend on ligand-dependent EGFR-mediated activation. The authors’ conclusions were that many TNBC may be EGFR pathway components dependent, but the constitutive pathway activation in many cases may not be via EGFR but by downstream components. The role of EGFR in BC has been reviewed by Masuda *et al*. A promising EGFR role as enhancer of chemosensitivity- rewiring the apoptotic signaling network- or to prevent metastasis-through migration and invasion control- have been reported in BL2 and mesenchymal-like subtypes [101].

A review on randomized clinical trials of TNBC patients treated with platinum-based plus targeted therapies was carried out [102]. The results of this review confirmed that the combination therapy has a limited impact on both PFS and OS. Whilst the review identified six subgroups that differ according to the specific response to the different target treatments, suggesting that therapeutic results and treatment outcomes can be improved, this is only possible through an appropriate selection of patients.

From the clinical and correlative studies, it is now clear that EGFR inhibition alone is unlikely to provide disease control in most TNBC; combination strategies targeting other components of the pathway and dedicated tissue-based studies are likely to be necessary. We also believe that a better selection of patients is crucial to improve the rate of response to treatment, as is the case with the assessment of the mutational status of EGFR in adenocarcinomas of the lung, and the valuation of the status panRAS and BRAF in colorectal tumors, in order to select oncogene addicted tumors and treat them in a rational manner. TKIs, such as erlotinib and gefitinib, have not been very effective in the treatment of BC as well [103]. Many phase II studies of EGFR-TKIs in metastatic BC show at most a 5% RR [101]. Erlotinib treatment is more effective in combination with other chemotherapeutic agents, including capecitabine and docetaxel. In a study designed to research the additive efficacy of erlotinib with capecitabine and docetaxel, the overall response rate was 67% [104]. Gefitinib and docetaxel combination demonstrated an active and generally well-tolerated regimen in women with metastatic BC, who have not been previously treated for metastatic disease [105]. Many studies provided a biological rationale to test anti-IGF-IR/InsR therapy in combination with chemotherapy in patients with TNBC [106]. Anti-IGF-IR/InsR therapy has being tested in TNBC. Litzenburger and colleagues tested the sensitivity of triple negative cell lines with IGF gene expression [106]. The IGF gene expression signature was present and reversed in three different models (cancer cell lines or xenografts) of TNBC treated with different anti-IGF-IR therapies. The IGF signature was present in TNBC and TNBC cell lines, which were especially sensitive to BMS-754807, and sensitivity was significantly correlated to the expression of the IGF gene signature. *In vitro* studies with BMS-754807 showed growth inhibition and, in combination with docetaxel, tumor regression, that was associated with reduced proliferation, increased apoptosis, and mitotic catastrophe. Strategies in targeting the IGF system are addressed to: (I) ligand; (II) receptors; (III) downstream molecules signaling pathway. In a review on insulin and IGFR signaling in BC, Yang and Yee [107] analyzed the reasons of failure of IGF-IR targeting monoclonal antibodies. Novel targeting opportunities are based on crosstalk that occurs between IGF-IRs and EGFRs [108], VEGFRs [109] and G protein-coupled receptor signaling systems (GPCRs) [110].

IGF/insulin signaling produces the activation of the intracellular networks PI3K/AKT and RAS/MAPK. Therefore, several key molecules along these pathways might be relevant targets, including the mammalian target of rapamycin (mTOR), a serine/threonine protein kinase, and the non-receptor tyrosine kinase Src. mTOR inhibitors disrupt the negative feedback of the receptors and enhance IGF/insulin signaling and subsequent PI3K/AKT activation [111]. Thus, mTOR inhibitors ultimately stimulate the IGF system [112]. Co-targeting IGF-IR and mTOR might result in enhanced clinical benefit compared to mTOR inhibitor monotherapy. Studies showed that dual inhibition of IGF-IR and mTOR improved antitumor activity *in vitro* and in breast and other cancers [113].

The results of clinical trials (NCT01220570, NCT01061788, NCT01122199) are expected to reveal the benefits of co-targeting EGFR and IGF-IR pathways and mTOR. Other small molecule inhibitors of the downstream pathways, such as PI3K inhibitor LY294002 [114], S6K1 inhibitor H89 [115], MAPK inhibitor U0126 [116] and dual PI3K/mTOR inhibitor NVP-BEZ235 [117] are currently in preclinical and clinical studies.

### 2.3. IM Subtype

The IM subtype is enriched with genes that characterize the immune signaling processes. They include: (I) immune cell signaling; (II) cytokine signaling; (III) antigen processing and presentation; (IV) signaling through core immune transduction pathways. Lehmann [40,41] suggested that these gene expressions are attributable to the tumor cells themselves, while Prat [19] considers them coming from the microenvironment. Immune signaling genes within the IM subtype, substantially overlap with a gene signature for medullary BC, which is a rare, distinct form of TNBC with a good prognosis [118]. The ability to inhibit the growth of these tumor cells by using immunoregulative cytokines, such as IL-10, TGF, interferon gamma, appears to be intriguing, but still entirely speculative. The role of immunity in BC was object of the last San Antonio Breast Cancer Symposium (SABCS). Patients with TNBC lesions containing high levels of tumor infiltrating lymphocytes, appear to have better prognosis than patients with low levels of white blood cells infiltrate. The German group presented data from the GeparSixto trial [23]; they observed a correlation between tumor infiltrating lymphocytes and response to neoadjuvant carboplatin-based chemotherapy.

This data is confirmed in a study presented in ASCO 2014; Vinayak *et al.* demonstrated that the density of stromal (sTILs) and intratumoral (iTILs) lymphocytes are predictive of a response to platinum based therapy and are significantly associated with TNBC subtypes, with the highest frequency in the IM subtype [119]. Many cancers, including TNBC, actively evade detection and eradication by the immune system by expressing proteins on their cell surface, which interact with T-cell inhibitory receptors such as CTLA-4 and PD-1 (programmed death 1). These two immune checkpoints are involved in peripheral tolerance and in the immune escape mechanisms during both chronic viral infections and cancer. CTLA-4 functions as an off switch to T-cell activity in the priming phase; PD-1 regulates T-cells activity during the effector phase and can shut down antigen-specific T-cell in the tumour microenvironment [120]. Recent evidence suggests that tumour cells can activate the PD-1 checkpoint, and then costitutively inactivate the T-cells, by expressing PD-L1 (programmed death ligand). PD-L1 has been discovered in a variety of epithelial cancer and also in BC [121]. A study presented by Barbara Pockaj at ASCO 2014 examined biomarkers involved in immune evasion including PD-L1 and its association with other biological pathways as potential treatment options for TNBC patients [122]. A subset of TNBC patients were found with elevated expressions of immune regolatory targets including PD-L1, CTLA-4 and IDO1. These observations provide a strong rationale for using antibodies capable of inhibiting these pathways in their treatment. In the study PD-L1 appears to be associated with AR negative TNBC and with BRCA1 mutated TNBC. There was an inverse correlation of BRCA1 with PD-L1 suggesting the use of platinum based therapy and PARP inhibitors in combination with anti PD-L1. Another finding was the positive correlation of PIK3CA and PD-L1, which may indicate there is an advantage to using a combination of therapies targeting both pathways. A number of different PD-1 and PD-L1 antibodies are currently being evaluated in clinical trials. For example, pembrolizumab is a humanized monoclonal IgG4 antibody directed against PD-1. It is under investigation in a phase I study including patients with advanced TNBC, advanced head and neck cancer, advanced urothelial cancer, or advanced gastric cancer (NCT01848834). Nivolumab is an anti PD-1 drug and is evaluated in combination with ipilimumab, an anti CTLA-4 drug, in a phase I/II trial for metastatic solid tumours including TNBC (NCT01928394). An ongoing phase 1b trial, using the PD-1 inhibitor lambrolizumab (MK-3475), includes 3 patient cohorts, one of which TNBC (NCT01848834).

Another variation on immunotherapy is targeting macrophages, to induce suppression of T-cells and cytokine secretion that promotes angiogenesis. Indeed, elevated numbers of macrophages have been found in residual tumors from patients who did not achieve a pCR from neoadjuvant chemotherapy [23]. Therefore targeting these cells has been proposed as a way to boost the natural immunosurveillance. Two trials are ongoing for testing combinations of chemotherapy with immunotherapy: (1) eribulin (a microtubule-targeting chemotherapy) with the “macrophage inhibitor” PLX3397 (CSF-1 inhibitor) is currently underway in an all-comers metastatic BC trial (NCT01596751); (2) an even broader study for solid tumors, including TNBC, of PLX3397 with weekly paclitaxel is also available (NCT01525602). Finally, another metabolic mechanism of immunosuppression, via the indoleamine 2,3-dioxygenase (IDO) pathway, able to state acquired peripheral tolerance, is being targeted in metastatic BC in combination with docetaxel, aiming to take advantage of the synergistic effect with chemotherapy. The IDO inhibitor being studied is indoximod (1-methyl-d-tryptophan) (NCT01191216).

### 2.4. M and MSL Subtypes

Mesenchymal-like subtypes (M and MSL subtypes) display a similar variety of unique gene ontology that is heavily enriched in those pathways which are involved in cell motility (focal adhesion, integrin signaling, Rac1, striated muscle contraction, and regulation of actin by Rho GTPase). Analysis of the mesenchymal-like subtypes also demonstrated enrichment in signaling pathways that are prominent in the processes of ETM (TGFβ, ECM-receptor interaction, ALK, Wnt/β-catenin, and mTOR, Rac1/Rho), decreased E-cadherin expression, ECM receptor interaction and cell differentiation pathways (Wnt pathway, anaplastic lymphoma kinase (ALK) pathway, and TGFβ signaling). Conversely, the M and MSL subtype differs in the expression of proliferation-associated gene. The M subtype displays higher expression of proliferation-associated genes, including Ki-67, while MSL subtype expresses low levels of them, along with an enrichment in the expression of genes associated with stem cells [40]. The signaling pathways expressed in the MSL groups share similar features to that highly dedifferentiated chemoresistant metaplastic BC, which may show both mesenchymal/sarcomatoid or squamous features [123]. A study found that 47% of metaplastic BC do show PIK3CA mutations with a high phosho-AKT expression [124].

TNBC mesenchymal-like cell lines preferentially responded to the dual PI3K/mTOR inhibitor NVP-BEZ235, that suggests the PI3K/mTOR pathway to be important in the mesenchymal-like subtype and a potentially therapeutic target. In addition, mutations in the Wnt/β-catenin pathway (CTNNB1, APC and WISP3) [125], that regulate EMT and may contribute to tumor cell invasion, occur frequently (52%) in metaplastic BC, suggesting that the deregulating Wnt/β-catenin pathway may be a viable therapeutic target in these tumors [126].

Inhibitors of Wnt/β-catenin are of great interest, currently in preclinical development [127]. Windorphen, a small molecule that selectively blocks the Wnt signal, exhibits remarkable specificity toward β-catenin-1 function and some anti-tumor activity, selectively killing cancer cells which harbor Wnt-activating mutations, so supporting the therapeutic potential role of this Wnt inhibitor class [128]. Drugs targeting this pathway could be of value for treating mesenchymal-like TNBC. However, unique to the MSL are genes representing both components and processes linked to growth factor signaling pathways. The MSL subtype is also uniquely enriched in genes involved in angiogenesis, as well as in immune signaling, which is evidenced by an overlap in GE unique to the IM subtype. Moreover, it displays low expression of claudins 3, 4 and 7, and, is made up, at least in part, by claudin-low tumors subtype of BC [10].

Hierarchical clustering of TNBC GE profiles, using the claudin-low gene predictor set (*n* = 770), segregated a portion of the M and MSL subtypes with low claudin. The diagnosis of these subtypes has clinical relevance. Patients with the M and MSL subtypes had decreased 5-year distant metastases-FS (DMFS), consistent with enrichment in pathways associated with metastasis and motility [39], also warns against the worst OS in the M subtype, despite its not so low rate of pCR. Interestingly, the M and MSL subtypes differed clinically, with patients in the M subtype presenting a shorter relapse-free survival (RFS), according to their high expression of genes of proliferation. The unique gene ontologies that characterize the M and MSL subtypes are to be expected, considering the specific treatment that can inhibit ETM, cell motility, angiogenesis and the pathway PI3K/AKT/mTOR. The non-receptor tyrosine kinase Src, a family member of Src kinase (SFK), plays many critical roles in cell migration, differentiation, motility, invasion, proliferation and cell survival, making it an ideal target for mesenchymal-like subtypes and leading to the investigation of Src-inhibitors. Src was found to be overexpressed or to have increased activity in breast cancer tumors [129]. In fact, the higher frequency of aberrant Src was observed in TNBC [130].

Lehmann [40,41] also showed that cell lines belonging to the mesenchymal-like subtypes are more sensitive than other subtype of TNBC cell lines to dasatinib, an oral small molecule which inhibits multiple kinase such as src and abl. Recently, dasatinib was approved for the treatment of imatinib refractory chronic myelogenous leukemia (CML) and bcr-abl positive acute lymphoblastic leukemia (ALL) [131]. *In vitro* studies demonstrated that dasatinib inhibits proliferation of triple negative cell lines. In the study of Finn *et al.* [132], caveolin-1 (CAV1), moesin (MSN), and yes-associated protein (YAP1) were identified as potential biomarkers of dasatinib sensitivity. A parallel independent study of Huang *et al.* [133], identified a six-gene panel that predicted sensitivity to dasatinib. These genes included EPHA2, CAV1, CAV2, ANXA1, PTRF and IGFBP2. All these genes are either targets for dasatinib or substrates for Src kinases. Many of these genes involved in cell migration, chemotaxis, ETM, adhesion, membrane remodeling, are expressed in M and MSL subtypes; and EPHA2l, that regulates cell adhesion and differentiation through DSG1/desmoglein-1 and inhibition of ERK1/ERK2 (MAPK3/MAPK1, respectively) signaling pathways is expressed in BL2 subtype [40]. A phase II trial (CA180059) [134] failed to demonstrate dasatinib activity when administered as monotherapy in patients with metastatic TNBC. In this study, 44 pretreated patients received dasatinib 100 mg twice daily. ORR was only 4.7%, and mPFS was 8.3 weeks. No grade 4 adverse events were reported while the most frequent grade 3 adverse events were fatigue, diarrhea, pleural effusion and dyspnea. A limitation of the study was an inadequate selection of patients without dasatinib activity biomarkers. Better results could be associated with dasatinib combination therapy. Kim *et al.* [135], published data from an *in vitro*-study: breast cancer cell lines were examinated for growth inhibition, apoptosis, cell migration and invasion after treatment with dasatinib, cetuximab and cisplatin alone or in combination. This study showed that dasatinib was effective in attenuating EGFR resistance in TNBC cell lines, leading to cell growth inhibition and apoptosis. A significant reduction in tumor cell migration and invasion was also found following dasatinib treatment, alone or in combination. Indeed, Src is a key substrate in the transduction pathways which is mediated by both EGFR and IGF-IR, as demonstrated by the inhibition of migration that is induced by EGFR/IGF-IR via a Src inhibitor in claudin-low cell lines [136]. Two phase I trials experimented the association of dasatinib with chemotherapy. The study of Fornier *et al.* [137], enroled 15 patients with metastatic BC, six of whom were triple negative. Patients received dasatinib in combination with weekly paclitaxel. The main side effects were hematological toxicity, edema and pleural effusion without any grade 4 adverse events. Preliminary activity data were encouraging: partial response and stable disease rate were respectively 31% and 29%. A phase I–II trial evaluating the association of dasatinib and paclitaxel in metastatic setting is now ongoing (CA180194). In the study of Somlo *et al.* [138] 25 metastatic BC patients treated with dasatinib and capecitabine, were able to be evaluated. 40% of patients were hormone-receptor (HR) negative. Clinical benefit was 56% while mPFS was 14.4 weeks.

EGFR has also been implicated as a key role player in the mitogenic and motogenic effects. Recent studies have shown that EGFR and IGF-IR regulate migration, tumor invasion and EMT. EGFR inhibitors induced a restoring from mesenchymal to epithelial phenotype in TNBC cells and the EGFR TKIs erlotinib inhibited tumor growth and metastasis in a SUM149 xenograft mouse model [139], showing an antimetastatic effect that could be the basis of “overlap sensitivity” to dasatinib between MSL and BL2 subtypes.

Despite the fact that EGF and IGF and Src have strong mitogenic and pro-migratory properties and promote metastasis, until today the strategy of targeting just one of them seems not to be enough to inhibit tumor behavior. So, the eventually gained clinical remission, in patients not selected on the basis of predictors of response, will be no more than transient, cause of the development of drug resistance.

The MSL subtype is also enriched in genes involved in angiogenesis. Proliferation and expansion of solid tumors are strictly related to neoangiogenesis. Thus, targeting and inhibiting blood vessels formations represents a promising therapeutic approach for M like subtypes. Agents that target angiogenesis are appealing for the treatment of TNBC because higher levels of vascular endothelial growth factor (VEGF) and VEGF-2 have been shown in women with TNBC, suggesting its potential as a prognostic tool as well as a putative target for therapeutic intervention [140].

Bevacizumab is a humanized monoclonal antibody which binds and neutralizes VEGF-A, a key mediator of angiogenesis, and has proved to be effective in colorectal cancer, non small cell lung cancer, renal cell carcinoma, ovarian carcinoma, and glioblastoma multiforme. However, the treatment with bevacizumab in combination with chemotherapy have been less successful in BC. After an initial FDA accelerated approval for first line treatment of HER2-negative metastatic BC, bevacizumab-paclitaxel indication was revoked, due to unconfirmed OS benefits. In contrast, bevacizumab plus paclitaxel or capecitabine continue to be recommended by EMA (European Medicine Agency) as first line treatment of metastatic BC.

One explanation for the lack of an overall survival benefit may be that BC is a clinically heterogeneous disease, with molecular subtypes responding differently to various treatments.

High baseline seric levels of VEGF in BC patients have been found to correlate significantly with increased OS, and were of borderline significance (*p* = 0.06) with improved PFS following bevacizumab treatment [141]. Furthermore, Yang *et al.* [142] demonstrated that patients with high expression of VEGF in the tumor cells, and CD31 and PDGFR in the tumor vasculature were more likely to respond to bevacizumab in combination with doxorubicin-docetaxel. TNBC is shown to have increased CD31 and VEGF expression suggesting that this subtype of tumors could have a beneficial effect from antiangiogenic therapy [143].

Besides, a study on BC xenografts demonstrated that basal-like and luminal-like models responded differently to antiangiogenic treatment in combination with chemotherapy *in vivo*, with clear improvements in basal-like when adding bevacizumab to doxorubicin and no benefit from the combination in luminal-like models [144]. The use of bevacizumab in patients with TNBC is supported by molecular and clinical data. 3 phase III trials analyzed the efficacy and the safety of bevacizumab-based chemotherapy in the subgroup of TNBC: E2100, AVADO, RIBBON 1. The E2100 study [145] showed that the addition of bevacizumab 10 mg/kg to weekly paclitaxel doubled PFS: in TNBC subgroup PFS was 10.6 months for the combination therapy, and 5.3 months for paclitaxel alone (HR 0.49), without any OS gain. The AVADO study [146] randomized patients to docetaxel either alone or in combination with bevacizumab. An unplanned subgroup analysis of the ER/PR/HER negative subset, revealed an mPFS of 8.2 and 5.4 months for combination therapy and chemotherapy alone, respectively. No OS benefit was demonstrated. In the RIBBON-1 trial bevacizumb *versus* placebo was administered in association with different schedules including anthracyclin/taxanes/capecitabine based chemotherapy [147].

In TNBC subgroup a non significant improvement in the mPFS was demonstrated in both the capecitabine (4.2 *versus* 6.1 months HR 0.72) and anthracycline/taxanes cohorts (8.2 *versus* 15.4 months HR 0.78). These data were confirmed by a metanalysis [148] including E2100, AVADO and RIBBON-1 trials. The analyzed population included 2447 patients, 621 from these having TNBC. In the subgroup of patients with TNBC, the PFS was 8.1 months and 5.4 months for bevacizumab and non-bevacizumab therapy, respectively. The HR was 0.63. Also RR was significantly higher with bevacizumab-containing therapy. No difference in OS between the two treatment arms was detected either for the overall population or for the subgroups of TNBC (HR 0.96 *p* = 0.67). Another metanalysis [149] of the 3 first line phase III studies and a Cochrane review, confirmed the improvement of ORR and PFS by bevacizumab combined chemotherapy, but no significant OS advantages.

The metanalysis interpretation is that even without OS improvement, bevacizumab should be used in TNBC as better alternatives are lacking, and the poor prognosis of these patients also needs considering. Unfortunately, TNBC tumors are considered in a comprehensive manner one more time, and any sub-analysis in the different subtypes of TNBC is not available aiming to more precisely evaluate the impact of treatment on PFS and OS.

As already discussed, PI3K/AKT/mTOR pathway appears to be enriched in the mesenchymal and LAR subtypes [40]. PI3K/AKT/mTOR pathway is frequently altereted in BC. Approximately 30% of BC have mutations in the PIK3CA gene and this is the most common mutation in TNBC [150]. Other mechanisms of activation of PI3K/AKT/mTOR pathway in TNBC are PTEN mutation, PIK3CA gene amplification, proteins phosphorylation [151]. A recent study shows that in residual tumors after standard anthracycline-taxane chemotherapy in TNBC patients, several PI3K pathway components are activated and correlate with relapse-free survival, suggesting this pathway to be therapeutically relevant [152]. Everolimus (RAD001) is an inhibitor of serine-threonine kinase mammalian target of rapamycin (mTOR). Several clinical trials have reported the effectiveness of everolimus when used in combination with trastuzumab or hormone therapy against HER2-overexpressing or hormone-receptor-overexpressing BC, respectively [153,154]. However, there are few studies which examinate the effects of everolimus against TNBC. Yunokova *et al.* [155], demonstrated everolimus activity in TNBC cell lines expressing EGFR or CK5/6, everolimus resistency in TNBC cell lines with cancer stem cells markers, such as decreased E-cadherin and increased expression of Snail or Twist. BEZ235 is a PI3K/mTOR dual inhibitor that has proven its ability to significantly reduce the growth of cancer cells with activating PI3K mutation [156]. *In vivo*, effect of BEZ235 on TNBC tumors was proved on conditional mouse model. The drug decreases tumor growth slowing cell cycle progression (cytostatic effect) when administered alone, while caused eradication of the tumor when combined with drugs which provoke cell death (cytotoxic effect), like carboplatin or taxotere [157].

In the study of Juvekar *et al.* [158] the PI3K inhibitor BKM120 was used in combination with the PARP inhibitor olaparib for the treatment of BRCA1-related BC in a mouse model. The two drugs had an *in vivo* synergistic activity, resulting in a tumor doubling time of over 70 days *versus* 26 and 16 days of BKM120 and olaparib alone, respectively. High sensitivity of BRCA1-mutant tumors to PI3K pathway inhibitor is the consequence of the role of the PI3K pathway in maintaining cell survival during DNA repair and in facilitating DNA repair mechanisms. There are three ongoing clinical trials experimenting the role of PI3K/AKT/mTOR inhibitors in TNBC.

One trial is a phase 2 trial of Merck’s AKT inhibitor (MK-2206) for advanced BC patients with PIK3CA or AKT mutation or PTEN alterations (NCT01277757). Buparlisib (BKM120), an oral pan-PI3K inhibitor is administered in combination with paclitaxel in a phase II trial including patients with HER2-negative, locally advanced or metastatic BC, with or without PI3K pathway activation (BELLE-4 trial) (NCT01790932). In a phase IB trial GDC-0941 is given in combination with paclitaxel, with or without bevacizumab (NCT00960960).

Even in the analysis of these trials in progress may be of great clinical relevance to stratify the results in the different subtypes, especially waiting for mesenchymal and LAR subtypes.

### 2.5. LAR Subtype

LAR genes ontology is heavily enriched in hormonally regulated pathways, such as steroid synthesis, porphyrin metabolism, and androgen/estrogen metabolism. Tumors in the LAR subtype display luminal GE patterns. The majority were classified as either luminal A or luminal B, none were classified as basal-like, further supporting the luminal origin of the LAR subtype [40,41].

LAR tumors are rare, the prevalence being 11% of TNBC and only 2% of all BC. Prat reported an incidence of luminal TNBC of 6.6% of the whole TNBC population [19]. Androgen receptor (AR) signaling, in ER-negative BC, could be responsible for the GE patterns in the LAR subtype. Lehmann [40,41] investigated AR protein expression by IHC in all TNBC tumors, showing that AR is espressed more and with greater intensity in LAR subtype as well as AR mRNA and several AR intracellular targets and coactivators. Similar data also arise from an unsupervised analysis that has identified a group of ER and PR negative but AR-expressing BC. This AR positive BC group is regulated at the transcriptional level by androgen and it expresses genes that characterize ER positive tumors [159].

Farmer [160] has described a BC subgroup expressing AR termed *molecular apocrine*. The GE profiles of all 6 apocrine tumors described in the Lehmann study [40,41] strongly correlate with LAR, all suggesting that the LAR-TNBC subtype is composed of AR-driven tumors that include the molecular apocrine subtype. Masuda [39] has reported that in neoadjuvant setting LAR tumors had a low (10%) pCR rate when treated with cytotoxic drugs, according to luminal profile. Indeed, the luminal A and B intrinsic subtypes, which are hormonally regulated tumors, showed a lower chemosensitivity [161,162].

Also the clinical outcomes of LAR subtype has a different clinical course from that of the other subtypes, and it is close to that of luminal tumors. Masuda reported that 75% of distant metastasis in the LAR subtype occurred more than three years after diagnosis, showing that the LAR group had delayed recurrences compared to the other groups which are characterized by early relapse of the disease [39]. Moreover, LAR subtype, despite having a low pCR rate compared to other subtypes, did not have the lowest OS rate, in agreement with the less severe prognosis of luminal BC. Lehmann [40,41] also reported that the RFS was significantly decreased in the LAR subtype compared to the BL1 (hazard ratio [Hr] = 2.9), IM (Hr = 3.2), while DMFS did not vary between TNBC subtypes (log-rank test; *p* = 0.2176), suggesting that recurrence is mainly related to locoregional relapse. Lehmann [40,41], after all, reported that LAR tumors do not differ for stage and grade compared to the other subtypes, while clinically differ for the more advanced age at the time of onset. These results, suggest that within TNBC we need to distinguish the LAR subtype and design a different treatment strategy for this group. Moreover, these findings suggest that the AR may serve as a therapeutic target for LAR subtype, similarly to prostate cancer. The AR protein is expressed at high levels in benign luminal/secretory prostate epithelial cells and in the vast majority of primary and metastatic prostate cancer cells. Prostate cancer is an androgen-dependent tumor in which androgen deprivation therapy continues to be the standard of care. The irreversible inhibitor of cytochrome CYP17, abiraterone, is being tested in a clinical trial still ongoing in AR expressing BC (NCT01842321) due to its proven efficacy in advanced castrated-resistant prostate cancers [163,164]. A similar phase II study, using in this case the AR inhibitor, bicalutamide, in patients with AR-positive BC, is currently underway (NCT00468715). Enzalutamide is an androgen receptor inhibitor that competes with AR binding and inhibits nuclear traslocation and its interaction with DNA. The drug has been got the approval in metastatic androgen resistant metastatic prostate cancer (AFFIRM [165] PREVAIL 13 [166]). A phase 2 clinical trial evaluating enzalutamide as single agent for the treatment of advanced, AR-positive, TNBC is ongoing (NCT01889238).

In analogy with prostate cancer it could be of great interest in cancer LAR subtype also to validate the antitumor activity of taxanes. In chemo- and castration resistant metastatic prostate cancer (mCRPC) docetaxel has been shown to improve OS and health-related quality of life [167,168], as well as the third-generation taxane cabazitaxel in mCRPC patient who have progressed after docetaxel-based chemotherapy [169].

Taxanes may be considered as a target agent by altering AR nuclear traffic and AR activity through microtubular stabilization and could play a significant role in the treatment of cancer LAR subtype, too. Since AR requires the Hsp90 chaperone for proper protein folding and stability [170], the LAR subtype could be sensitive to the Hsp90 inhibitors. Moreover, the LAR cell lines were more sensitive to the Hsp90 inhibitor, 17-dimethylaminoethylamino-17-demethoxygeldanamycin (17-DMAG). Heat shock proteins (Hsp) are a group of molecules responsible for controlling the correct folding, stability and function of numerous important signaling proteins involved in cell growth, differentiation and survival [171,172]. Among them, Hsp90 is the most abundant molecular chaperone found in mammalian cells, widely expressed in BC [173]. Hsp90 acts as part of a multichaperone complex, that includes other co-chaperones, such as Hsp70, p23 and Hop, and interacts with a variety of proteins that play key roles in cancer progression, including ErbB2, Bcr-Abl, Akt, B-Raf, C-Raf, CDK4, PLK-1, MET, mutated p53, HIF-1, steroid hormone receptors (oestrogen and androgen), survivin and telomerase hTERT [174,175]. Five isoforms of Hsp90 have been identified, which have different cellular localizations. Hsp90a and Hsp90b are the two major cytoplasmic isoforms, which share about 85% of aminoacid sequence, Grp94 is the isoform which is present predominantly in the endoplasmic reticulum, TRAP1/HSP75 stays in the mitochondrial matrix, and HSP90N is associated with cellular transformation [174]. It has been reported that Hsp90 is usually overexpressed in different human tumors, where it is not mutated or indeed amplified [174]. Since these proteins act as molecular chaperones either helping in the refolding of misfolded proteins or in their degradation, recent studies reported that Hsp90 may be a clinical biomarker or molecular target for cancer therapy. This has led to the development of several molecules able to inhibit Hsp90, including geldanamycin (GA), a benzoquinone ansamycin antibiotic, that was excluded from clinical trials for its liver toxicity profile, and 17-(allylamino)-17-demethoxygeldanamycin (17-AAG) (tanespimycin) and 17-DMAG, which differ from GA, just in their 17-substituents. 17AAG and 17DMAG are proving to be well tolerated and suitable for further evaluation, as well as a number of other small molecule inhibitors [176,177,178,179,180,181] and are now entering phase II clinical trials.

Strong and durable anti-tumor effects in TNBC xenografts, including complete response and tumor regression without toxicity to the host, are achieved by PU-H71-bound Hsp90. PU-H71 induces: (I) efficient and sustained downregulation, both *in vitro* and *in vivo*, of components of the Ras/Raf/MAPK pathway with anti-proliferative effect; (II) degradation of activated Akt and Bcl-xL leading to apoptosis; (III) inhibition of activated NF-κB, Akt, ERK2, Tyk2, and PKC impairing the TNBC invasive potential. The results identify Hsp90 as a critical and multimodal target in TNBC and justifies the use of the Hsp90 inhibitor PU-H71 in clinical trials of patients with TNBC [182].

The antitumoral and anti-metastatic activity of ganetespib (STA-9090), a synthetic small-molecule inhibitor of Hsp90 was investigated using TNBC cell lines and xenograft models [183]. Ganetespib simultaneously deactivated multiple oncogenic pathways to highly reducing cell viability in TNBC cell lines, and suppressed lung metastases in experimental models. Ganetespib potentiated the cytotoxic activity of doxorubicin also promoting mitotic catastrophe and apoptosis in combination with taxanes *in vitro*, and significantly improved combinatorial activity *in vivo*. Marked tumor shrinkage was seen in patients under ganetespib monotherapy [184,185]. An open-label multicenter phase II study is recruiting patients with HER2-positive BC and TNBC for ganetespib treatment in first line (NCT01677455).

LAR cell lines which carry activating PIK3CA mutations were also sensitive to PI3K inhibitor NVP-BEZ235, as well as ER positive BC [151,184]. Also, the dual blockage of AR and PI3K/mTOR pathways may be synergistic in LAR TNBC as well as in AR-dependent prostate cancer cells [155].

## 3. Conclusions

**Figure 1:**
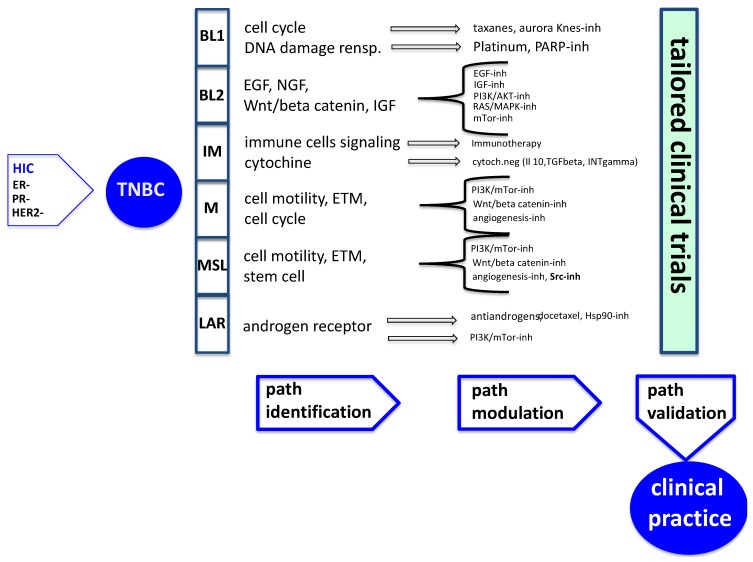
Optimize therapeutic strategies in metastatic TNBC. Schematic representation of the steps which are needed for a rational treatment of each subtype. The paths must be identified, modulated, and finally validate from small taloired clinical trials, in order to approve their acceptance in clinical practice.

Although considerable progress has been made in understanding the genetic targets in TNBC, many questions remain unanswered and many goals still to be achieved in order to optimize treatment strategies and ultimately improve the prognosis of patients with TNBC (Figure 1).

There is strong evidence that the failure to express ER, PR, as well as HER2, by BC cells on the base of their IHC-assessment, is only able to exclude molecular subtype luminal A, luminal B, HER2-enriched, but it does not identify an intrinsic molecular subtype of BC, although the majority of TNBC tumors phenotypically fall in basal-like molecular subtype.

It is clear that TNBC consists of a heterogeneous group of cancer, which differs for incidence, molecular characteristics, clinical course, response to therapy as well as prognosis.

There is evidence that the classification of TNBC in molecular subtype is able to identify these different diseases, with potential prognostic and therapeutic implications again. There is little doubt that the success of the treatment of TNBC will require the identification of genetic targets characteristic of each subtype at sustainable cost.

The IHC assay, which is technically simple and reproducible and that can be performed on paraffin-embedded tissue, seems to be the ideal method to evaluate the expression and the levels of key proteins that identify each subtype. IHC also allows selection of cases to be submitted to the fluorescence in situ hybridization FISH analysis, that is able to highlight electively amplifications, mutations and gene rearrangements. The identification of predictive markers of efficacy and toxicity is also a crucial issue.

There is little doubt that successful treatment of TNBC will require manipulation of genetic targets. Classification into subtypes enables the identification of potential therapeutic targets that are specific to each molecular subtype. Setting the therapeutic targets will allow the following: (I) the identification of chemosensitive subtypes in order to treat in a targeted manner those subtypes which are heavily enriched in cell cycle, which means to use such antimitotic agents as taxanes, as well as the use of some old drugs in a new way such as cisplatin, cyclophosphamide and mitomycin, possibly in association with PARP-inhibitors or AURORA-kinases, aiming to exploit the aberrant DNA repair and genome-wide instability of subtype BL1; (II) the identification of the subtype hormone-sensitive in order to use anti-androgens targeted therapies in LAR subtype, that can be rationally indicated only in well-selected patients based on the expression of the receptor; (III) the selection of chemoresistant tumours “oncogene addicted” and their treatment according to the specific alteration of the molecular patways.

Proper selection could confirm the positive results that generally emerge on the activities of a drug *in vitro*, generally not confirmed in phase II and III studies for lack of selection or for the impact by the microenviroment or tumor heterogeneity. Nevertheless, the experience gained from failures in recent years shows that the tumor cells respond to inhibition of a pathway by adapting their signaling circuits, taking advantage of redundant paths and feedback mechanisms and by cross-talking, in order to maintain their function. So, the eventually gained clinical remission will be no more than transient, cause of the development of drug resistance.

Particularly intriguing in TNBC, are novel strategies regarding the chaperon protein, which is able to inhibit multiple pathways irrespective of subsequent resistancy mutations, as well as immunotherapy. It is potentially able to transform subsequent meccanisms of resistance to memory, and may actually overcome drug resistance, which is the significant problem, with the target therapies as well as with the conventional ones.

The clinical use of setting the subtypes will not reach its full potential until almost one strategy approach for each subtype is not clinically validated by clinical trials; this will allow a rational treatment according to the specific objectives that guide the tumorigenicity, progression and metastatic potential of each TNBC subtype.

## Abbreviations

BL1: basal-like 1
BL2: basal-like 2
IM: immunomodulatory
M: mesenchymal
MSL: mesenchymal stem-like
LAR: luminal androgen receptor
AURKA: aurora kinase A
AURKB: aurora kinase B
MYC: myelocytomatosis oncogene
NRAS: neuroblastoma RAS (Rat sarcoma) viral (v-ras) oncogene homolog
DNA: deoxyribonucleic acid
ATR: ataxia telangiectasia and Rad3-related
BRCA: breast cancer susceptibility protein
RNA: ribonucleic acid
EGF: epidermal growth factor
IGF1R: insulin-like growth factor 1 receptor
MET: *N*-methyl-*N*’-nitroso-guanidine human osteosarcoma transforming gene
NGF: nerve growth factor
WNT: wingless-related integration site
TH1/TH2: helper T 1/helper T 2 cells
DC: dendritic cell
NK: natural killer
NFκB: nuclear factor kappa-light-chain-enhancer of activated B cells
TNF: tumor necrosis factor
JAK: Janus kinase
STAT: signal transducers and activators of transcription
CTL4: cytotoxic T-lymphocyte antigen 4
IL12: interleukin 12
IL7: interleukin 7
RHO: Ras homologous
ALK: anaplastic lymphoma kinase
TGFβ: transforming growth factor beta
IGF: insulin-like growth factor
mTOR: mammalian target of rapamycin
ECM: extracellular matrix
RAC1: Ras-related C3 botulinum toxin substrate 1
EMT: epithelial mesenchymal transition
PDGF: platelet-derived growth factor
GPCR: G protein-coupled receptor
ERK1/2: extracellular-signal-regulated kinases 1/2
ABC transporter: ATP-binding cassette transporter
PI3K: phosphatidylinositol 3-kinase
AKT: protein kinase B
HSP90: heat shock protein 90
PARP: Poly (ADP-ribose) polymerase
CSF-1: colony stimulating factor 1
IDO: indoleamine 2,3-dioxygenase
pCR: pathological complete response
CT: chemotherapy

## References

[1] Foulkes W.D., Smith I.E., Reis-Filho J.S. (2010). Triple-negative breast cancer. N. Engl. J. Med..

[2] Sasa M., Bando Y., Takahashi M., Hirose T., Nagao T. (2008). Screening for basal marker expression is necessary for decision of therapeutic strategy for triple-negative breast cancer. J. Surg. Oncol..

[3] Viale G., Rotmensz N., Maisonneuve P., Bottiglieri L., Montagna E., Luini A., Veronesi P., Intra M., Torrisi R., Cardillo A. (2009). Invasive ductal carcinoma of the breast with the “triple-negative” phenotype: Prognostic implications of EGFR immunoreactivity. Breast Cancer Res. Treat..

[4] Cheang M.C., Voduc D., Bajdik C., Leung S., McKinney S., Chia S.K., Perou C.M., Nielsen T.O. (2008). Basal-like breast cancer defined by five biomarkers has superior prognostic value than triple-negative phenotype. Clin. Cancer Res..

[5] Bostrom P., Söderström M., Palokangas T., Vahlberg T., Collan Y., Carpen O., Hirsimäki P. (2009). Analysis of cyclins A, B1, D1 and E in breast cancer in relation to tumour grade and other prognostic factors. BMC Res. Notes.

[6] Voduc D., Nielsen T.O., Cheang M.C., Foulkes W.D. (2008). The combination of high cyclin E and Skp2 expression in breast cancer is associated with a poor prognosis and the basal phenotype. Hum. Pathol..

[7] Geyer F.C., Lacroix-Triki M., Savage K., Arnedos M., Lambros M.B., MacKay A., Natrajan R., Reis-Filho J.S. (2011). β-Catenin pathway activation in breast cancer. Mod. Pathol..

[8] Rakha E.A., El-Sayed M.E., Green A.R., Lee A.H., Robertson J.F., Ellis I.O. (2007). Prognostic markers in triple-negative breast cancer. Cancer.

[9] Perou C.M., Sørlie T., Eisen M.B., van de Rijn M., Jeffrey S.S., Rees C.A., Pollack J.R., Ross D.T., Johnsen H., Akslen L.A. (2000). Molecular portraits of human breast tumours. Nature.

[10] Prat A., Parker J.S., Karginova O., Fan C., Livasy C., Herschkowitz J.I., He X., Perou C.M. (2010). Phenotypic and molecular characterization of the claudin-low intrinsic subtype of breast cancer. Breast Cancer Res..

[11] Perou C.M. (2011). Molecular stratification of triple-negative breast cancers. Oncologist.

[12] Nielsen T.O., Hsu F.D., Jensen K., Cheang M., Karaca G., Hu Z., Hernandez-Boussard T., Livasy C., Cowan D., Dressler L. (2004). Immunohistochemical and clinical characterization of the basal-like subtype of invasive breast carcinoma. Clin. Cancer Res..

[13] Bertucci F., Finetti P., Cervera N., Esterni B., Hermitte F., Viens P., Birnbaum D. (2008). How basal are triple-negative breast cancers?. Int. J. Cancer.

[14] Kreike B., van Kouwenhove M., Horlings H., Weigelt B., Peterse H., Bartelink H., van de Vijver M.J. (2007). Gene expression profiling and histopathological characterization of triple-negative/basal-like breast carcinomas. Breast Cancer Res..

[15] Prat A., Perou C.M. (2011). Deconstructing the molecular portraits of breast cancer. Mol. Oncol..

[16] Martín M., Rodríguez-Lescure A., Ruiz A., Alba E., Calvo L., Ruiz-Borrego M., Munárriz B., Rodríguez C.A., Crespo C., de Alava E. (2008). Randomized phase 3 trial of fluorouracil, epirubicin, and cyclophosphamide alone or followed by Paclitaxel for early breast cancer. J. Natl. Cancer Inst..

[17] Bramwell V.H.C., Pritchard K.I., Tu D., Tonkin K., Vachhrajani H., Vandenberg T.A., Robert J., Arnold A., O’Reilly S.E., Graham B. (2010). A randomized placebo-controlled study of tamoxifen after adjuvant chemotherapy in premenopausal women with early breast cancer (National Cancer Institute of Canada—Clinical Trials Group Trial, MA.12). Ann. Oncol..

[18] Levine M.N., Pritchard K.I., Bramwell V.H.C., Shepherd L.E., Tu D., Paul N. (2005). Randomized trial comparing cyclophosphamide, epirubicin, and fluorouracil with cyclophosphamide, methotrexate, and fluorouracil in premenopausal women with node-positive breast cancer: Update of National Cancer Institute of Canada Clinical Trials Group Trial MA5. J. Clin. Oncol..

[19] Prat A., Adamo B., Cheang M.C., Anders C.K., Carey L.A., Perou C.M. (2013). Molecular characterization of basal-like and non-basal-like triple-negative breast cancer. Oncologist.

[20] Morris G.J., Naidu S., Topham A.K., Guiles F., Xu Y., McCue P., Schwartz G.F., Park P.K., Rosenberg A.L., Brill K. (2007). Differences in breast carcinoma characteristics in newly diagnosed African-American and Caucasian patients: A single-institution compilation compared with the National Cancer Institute’s Surveillance, Epidemiology, and End Results database. Cancer.

[21] Carey L.A., Perou C.M., Livasy C.A., Dressler L.G., Cowan D., Conway K., Karaca G., Troester M.A., Tse C.K., Edmiston S. (2006). Race, breast cancer subtypes, and survival in the Carolina breast cancer study. JAMA.

[22] Tavassoli F.A., Devilee P. (2003). World Health Organization classification of tumours. Pathology and Genetics of Tumours of the Breast and Female Genital Organs.

[23] Denkert C., Loibl S., Salat C., Sinn B.V., Schem C., Endris V., Klare P., Schmitt W.D., Blohmer J.-U., Weichert W. (2013). Increased tumor-associated lymphocytes predict benefit from addition of carboplatin to neoadjuvant therapy for triple-negative and HER2-positive early breast cancer in the GeparSixto trial (GBG 66). Cancer Res..

[24] Atchley D.P., Albarracin C.T., Lopez A., Valero V., Amos C.I., Gonzalez-Angulo A.M., Hortobagyi G.N., Arun B.K. (2008). Clinical and pathologic characteristics of patients with BRCA-positive and BRCA-negative breast cancer. J. Clin. Oncol..

[25] Gonzalez-Angulo A.M., Timms K.M., Liu S., Chen H., Litton J.K., Potter J., Lanchbury J.S., Stemke-Hale K., Hennessy B.T., Arun B.K. (2011). Incidence and outcome of BRCA mutations in unselected patients with triple receptor-negative breast cancer. Clin. Cancer Res..

[26] Turner N., Tutt A., Ashworth A. (2004). Hallmarks of “BRCAness” in sporadic cancers. Nat. Rev. Cancer.

[27] Turner N.C., Reis-Filho J.S., Russell A.M., Springall R.J., Ryder K., Steele D., Savage K., Gillett C.E., Schmitt F.C., Ashworth A. (2007). BRCA1 dysfunction in sporadic basal-like breast cancer. Oncogene.

[28] Davis A.A., Kaklamani V.G. (2012). Metabolic syndrome and triple-negative breast cancer: A new paradigm. Int. J. Breast Cancer.

[29] Jardé T., Perrier S., Vasson M.P., Caldefie-Chézet F. (2011). Molecular mechanisms of leptin and adiponectin in breast cancer. Eur. J. Cancer.

[30] Eliassen A.H., Missmer S.A., Tworoger S.S., Spiegelman D., Barbieri R.L., Dowsett M., Hankinson S.E. (2006). Endogenous steroid hormone concentrations and risk of breast cancer among premenopausal women. J. Natl. Cancer Inst..

[31] Missmer S.A., Eliassen A.H., Barbieri R.L., Hankinson S.E. (2004). Endogenous estrogen, androgen, and progesterone concentrations and breast cancer risk among postmenopausal women. J. Natl. Cancer Inst..

[32] Singh A., Hamilton-Fairley D., Koistinen R., Seppälä M., James V.H., Franks S., Reed M.J. (1990). Effect of insulin-like growth factor-type I (IGF-I) and insulin on the secretion of sex hormone binding globulin and IGF-I binding protein (IBP-I) by human hepatoma cells. J. Endocrinol..

[33] Shimizu C., Hasegawa T., Tani Y., Takahashi F., Takeuchi M., Watanabe T., Ando M., Katsumata N., Fujiwara Y. (2004). Expression of insulin-like growth factor 1 receptor in primary breast cancer: Immunohistochemical analysis. Hum. Pathol..

[34] Davison Z., de Blacquière G.E., Westley B.R., May F.E. (2011). Insulin-like growth factor-dependent proliferation and survival of triple-negative breast cancer cells: Implications for therapy. Neoplasia.

[35] Carey L.A. (2010). Directed therapy of subtypes of triple-negative breast cancer. Oncologist.

[36] Liedtke C., Mazouni C., Hess K.R., André F., Tordai A., Mejia J.A., Symmans W.F., Gonzalez-Angulo A.M., Hennessy B., Green M. (2008). Response to neoadjuvant therapy and long-term survival in patients with triple-negative breast cancer. J. Clin. Oncol..

[37] Dent R., Trudeau M., Pritchard K.I., Hanna W.M., Kahn H.K., Sawka C.A., Lickley L.A., Rawlinson E., Sun P., Narod S.A. (2007). Triple-negative breast cancer: Clinical features and patterns of recurrence. Clin. Cancer Res..

[38] Smid M., Wang Y., Zhang Y., Sieuwerts A.M., Yu J., Klijn J.G., Foekens J.A., Martens J.W. (2008). Subtypes of breast cancer show preferential site of relapse. Cancer Res..

[39] Masuda H., Baggerly K.A., Wang Y., Zhang Y., Gonzalez-Angulo A.M., Meric-Bernstam F., Valero V., Lehmann B.D., Pietenpol J.A., Hortobagyi G.N. (2013). Differential response to neoadjuvant chemotherapy among 7 triple-negative breast cancer molecular subtypes. Clin. Cancer Res..

[40] Lehmann B.D., Bauer J.A., Chen X., Sanders M.E., Chakravarthy A.B., Shyr Y., Pietenpol J.A. (2011). Identification of human triple-negative breast cancer subtypes and preclinical models for selection of targeted therapies. J. Clin. Investig..

[41] Lehmann B.D., Pietenpol J.A. (2014). Identification and use of biomarkers in treatment strategies for triple-negative breast cancer subtypes. J. Pathol..

[42] Bauer J.A., Chakravarthy A.B., Rosenbluth J.M., Mi D., Seeley E.H., de Matos Granja-Ingram N., Olivares M.G., Kelley M.C., Mayer I.A., Meszoely I.M. (2010). Identification of markers of taxane sensitivity using proteomic and genomic analyses of breast tumors from patients receiving neoadjuvant paclitaxel and radiation. Clin. Cancer Res..

[43] Harris L.N., Broadwater G., Lin N.U., Miron A., Schnitt S.J., Cowan D., Lara J., Bleiweiss I., Berry D., Ellis M. (2006). Molecular subtypes of breast cancer in relation to paclitaxel response and outcomes in women with metastatic disease: Results from CALGB 9342. Breast Cancer Res..

[44] Rugo H.S., Barry W.T., Moreno-Aspitia A., Lyss A.P., Cirrincione C., Mayer E.L., Naughton M., Layman R.M., Lisa A., Carey L.A. (2012). CALGB 40502/NCCTG N063H: Randomized phase III trial of weekly paclitaxel (P) compared to weekly nanoparticle albumin bound nab-paclitaxel (NP) or ixabepilone (Ix) with or without bevacizumab (B) as first-line therapy for locally recurrent or metastatic breast cancer (MBC). J. Clin. Oncol..

[45] Byrski T., Huzarski T., Dent R., Gronwald J., Zuziak D., Cybulski C., Kladny J., Gorski B., Lubinski J., Narod S.A. (2008). Response to neo-adjuvant chemotherapy in women with BRCA1-positive breast cancers. Breast Cancer Res. Treat..

[46] Wysocki P.J., Korski K., Lamperska K., Zaluski J., Mackiewicz A. (2008). Primary resistance to docetaxel-based chemotherapy in metastatic breast cancer patients correlates with a high frequency of BRCA1 mutations. Med. Sci. Monit..

[47] Thompson L.H., Schild D. (2001). Homologous recombinational repair of DNA ensures mammalian chromosome stability. Mutat. Res..

[48] Dantzer F., de la Rubia G., Menissier-De Murcia J., Hostomsky Z., de Murcia G., Schreiber V. (2000). Base excision repair is impaired in mammalian cells lacking Poly(ADP-ribose) polymerase-1. Biochemistry.

[49] Bhattacharyya A., Ear U.S., Koller B.H., Weichselbaum R.R., Bishop D.K. (2000). The breast cancer susceptibility gene BRCA1 is required for subnuclear assembly of Rad51 and survival following treatment with the DNA cross-linking agent cisplatin. J. Biol. Chem..

[50] Moynahan M.E., Cui T.Y., Jasin M. (2001). Homology-directed DNA repair, mitomycin-c resistance, and chromosome stability is restored with correction of a Brca1 mutation. Cancer Res..

[51] Quinn J.E., Kennedy R.D., Mullan P.B., Gilmore P.M., Carty M., Johnston P.G., Harkin D.P. (2003). BRCA1 functions as a differential modulator of chemotherapy-induced apoptosis. Cancer Res..

[52] Bergamaschi A., Kim Y.H., Wang P., Sørlie T., Hernandez-Boussard T., Lonning P.E., Tibshirani R., Børresen-Dale A.L., Pollack J.R. (2006). Distinct patterns of DNA copy number alteration are associated with different clinicopathological features and gene-expression subtypes of breast cancer. Genes Chromosomes Cancer.

[53] Byrski T., Gronwald J., Huzarski T., Grzybowska E., Budryk M., Stawicka M., Mierzwa T., Szwiec M., Wisniowski R., Siolek M. (2010). Pathologic complete response rates in young women with BRCA1-positive breast cancers after neoadjuvant chemotherapy. J. Clin. Oncol..

[54] Silver D.P., Richardson A.L., Eklund A.C., Wang Z.C., Szallasi Z., Li Q., Juul N., Leong C.O., Calogrias D., Buraimoh A. (2010). Effıcacy of neoadjuvant cisplatin in triple-negative breast cancer. J. Clin. Oncol..

[55] Decatris M.P., Sundar S., O’Byrne K.J. (2004). Platinum-based chemotherapy in metastatic breast cancer: Current status. Cancer Treat. Rev..

[56] Carey L.A., Dees E.C., Sawyer L., Gatti L., Moore D.T., Collichio F., Ollila D.W., Sartor C.I., Graham M.L., Perou C.M. (2007). The triple negative paradox: Primary tumor chemosensitivity of breast cancer subtypes. Clin. Cancer Res..

[57] Baselga J., Gomez P., Awada A. (2010). The addition of cetuximab to cisplatin increases overall response rate and progression-free survival in metastatic triple-negative breast cancer: Results of a randomized phase II study (BALI-1). Ann. Oncol..

[58] Isakoff S.J., Goss P.E., Mayer E.L., Traina T.A., Carey L.A., Krag K., Rugo H.S., Liu M.C., Stearns V., Come S.E. (2011). TBCRC009: A multicenter phase II study of cisplatin or carboplatin for metastatic triple-negative breast cancer and evaluation of p63/p73 as a biomarker of response. J. Clin. Oncol..

[59] Maisano R., Zavettieri M., Azzarello D., Raffaele M., Maisano M., Bottari M., Nardi M. (2011). Carboplatin and gemcitabine combination in metastatic triple-negative anthracycline- and taxane-pretreated breast cancer patients: A phase II study. J. Chemother..

[60] Wang Z., Hu X., Chen L., Wang J., Wang H., Wang L., Liu G., Hu Z., Wu J., Zhimin S. (2010). Efficacy of gemcitabine and cisplatin (GP) as first-line combination therapy in patients with triple-negative metastatic breast cancer: Preliminary results report of a phase II trial. J. Clin. Oncol..

[61] Kim T., Lee H., Han S., Oh D., Im S., Bang Y. (2010). The comparison of the benefits obtained from platinum-containing chemotherapy between triple-negative and non-triple-negative metastatic breast cancer. J. Clin. Oncol..

[62] Liu M., Mo Q.G., Wei C.Y., Qin Q.H., Huang Z., He J. (2013). Platinum-based chemotherapy in triple-negative breast cancer: A meta-analysis. Oncol. Lett..

[63] Isakoff S.J., He L., Mayer E.L., Goss P.E., Traina T.A., Carey L.A., Krag K., Liu M.C., Rugo H.S., Stearns V. (2014). Identification of biomarkers to predict response to single-agent platinum chemotherapy in metastatic triple-negative breast cancer (mTNBC): Correlative studies from TBCRC009. J. Clin. Oncol..

[64] Leong C.O., Vidnovic N., Deyoung M.P., Sgroi D., Ellisen L.W. (2007). The p63/p73 network mediates chemosensitivity to cisplatin in a biologically defined subset of primary breast cancers. J. Clin. Investig..

[65] Hamilton E., Kimmick G., Hopkins J., Marcom P.K., Rocha G., Welch R., Broadwater G., Blackwell K. (2013). nNab-paclitaxel/bevacizumab/carboplatin chemotherapy in first-line triple negative metastatic breast cancer. Clin. Breast Cancer.

[66] Baselga J., Stemmer S., Pego A., Chan A., Goeminne A.J., Graas C.M., Kennedy P.J., Ciruelos Gil E.M., Zubel A., Groos J. (2010). Cetuximab+ cisplatin in estrogen receptor-negative, progesterone receptor-negative, HER2-negative (triple-negative) metastatic breast cancer: Results of the randomized phase II BALI-1 trial. Cancer Res..

[67] Sharma P., Khan Q.J., Kimler B.F., Klemp J.R., Connor C.S., McGinness M.K., Mammen J.W.M., Tawfik O.W., Fan F., Fabian C.J. (2010). Results of a phase II study of neoadjuvant platinum/taxane based chemotherapy and erlotinib for triple negative breast cancer. Cancer Res..

[68] O’Shaughnessy J., Osborne C., Pippen J.E., Yoffe M., Patt D., Rocha C., Koo I.C., Sherman B.M., Bradley C. (2011). Iniparib plus chemotherapy in metastatic triple-negative breast cancer. N. Engl. J. Med..

[69] Carey L.A., Rugo H.S., Marcom P.K., Irvin W., Ferraro M., Burrows E., He X., Perou C.M. (2008). TBCRC 001: EGFR inhibition with cetuximab added to carboplatin in metastatic triple-negative (basal-like) breast cancer. J. Clin. Oncol..

[70] Tutt A., Ashworth A. (2002). The relationship between the roles of BRCA genes in DNA repair and cancer predisposition. Trends Mol. Med..

[71] Byrski T., Huzarski T., Dent R., Gronwald J., Zuziak D., Cybulski C., Kladny J., Gorski B., Lubinski J., Narod S.A. (2009). Response to neoadjuvant therapy with cisplatin in BRCA1-positive breast cancer patients. Breast Cancer Res. Treat..

[72] Farmer H., McCabe N., Lord C.J., Tutt A.N., Johnson D.A., Richardson T.B., Santarosa M., Dillon K.J., Hickson I., Knights C. (2005). Targeting the DNA repair defect in BRCA mutant cells as a therapeutic strategy. Nature.

[73] Tentori L., Portarena I., Graziani G. (2002). Potential clinical applications of poly(ADP-ribose) polymerase (PARP) inhibitors. Pharmacol. Res..

[74] He J.X., Yang C.H., Miao Z.H. (2010). Poly(ADP-ribose) polymerase inhibitors as promising cancer therapeutics. Acta Pharmacol. Sin..

[75] Tutt A., Robson M., Garber J.E., Domchek S.M., Audeh M.W., Weitzel J.N., Friedlander M., Arun B., Loman N., Schmutzler R.K. (2010). Oral poly(ADP- ribose) polymerase inhibitor olaparib in patients with BRCA1 or BRCA2 mutations and advanced breast cancer: A proof-of- concept trial. Lancet.

[76] O’Shaughnessy J., Schwartzberg L.S., Danso M.A., Rugo H.S., Miller K., Yardley D.A., Carlson R.W., Finn R.S., Charpentier E., Freese M. (2011). A randomized phase III study of iniparib (BSI-201) in combination with gemcitabine/carboplatin (G/C) in metastatic triple negative breast cancer (TNBC). J. Clin. Oncol..

[77] Yang S.X., Kummar S., Steinberg S.M., Murgo A.J., Gutierrez M., Rubinstein L., Nguyen D., Kaur G., Chen A.P., Giranda V.L. (2009). Immunohistochemical detection of poly(ADP-ribose) polymerase inhibition by ABT-888 in patients with refractory solid tumors and lymphomas. Cancer Biol. Ther..

[78] Donawho C.K., Luo Y., Luoetal Y. (2007). ABT-888, anorallyactive poly(ADP-ribose) polymerase inhibitor that potentiates DNA-damaging agents in preclinical tumor models. Clin. Cancer Res..

[79] Isakoff S.J., Overmoyer B., Tung N.M., Gelman R.S., Giranda V.L., Bernhard K.M., Habin K.R., Ellisen L.W., Winer E.P., Goss P.E. (2010). A phase II trial of the PARP inhibitor veliparib (ABT888) and temozolomide for metastatic breast cancer. J. Clin. Oncol..

[80] Rugo H., Olopade O., de Michele A., van’t Veer L., Buxton M., Hylton N., Yee N., Chien D.M.J., Wallace A. (2013). Veliparib/carboplatin plus standard neoadjuvant therapy for high-risk breast cancer: First efficacy results from the I-SPY 2 TRIAL. Cancer Res..

[81] Somlo G., Frankel P.H., Luu T.H., Ma C., Arun B., Garcia A., Cigler T., Cream L., Harvey H.A., Sparano J.A. (2014). Phase II trial of single agent PARP inhibitor ABT-888 (veliparib [vel]) followed by postprogression therapy of vel with carboplatin (carb) in patients (pts) with stage BRCA-associated metastatic breast caner (MBC): California Cancer Consortium trial PHII-96. J. Clin. Oncol..

[82] Mina L.A., Ramanathan R.K., Wainberg Z.A. BMN 673 is a PARP inhibitor in clinical development for the treatment of breast cancer patients with deleterious germline BRCA 1 and 2 mutations. Proceedings of the SABCS.

[83] Fu J., Bian M., Jiang Q., Zhang C. (2007). Roles of Aurora kinases in mitosis and tumorigenesis. Mol. Cancer Res..

[84] Marumoto T., Zhang D., Saya H. (2005). Aurora-AA guardian of poles. Nat. Rev. Cancer.

[85] Giet R., Petretti C., Prigent C. (2005). Aurora kinases, aneuploidy and cancer, a coincidence or a real link?. Trends Cell Biol..

[86] Sasai K., Katayama H., Stenoien D.L., Fujii S., Honda R., Kimura M., Okano Y., Tatsuka M., Suzuki F., Nigg E.A. (2004). Aurora-C kinase is a novel chromosomal passenger protein that can complement Aurora-B kinase function in mitotic cells. Cell Motil. Cytoskelet..

[87] Yan X., Cao L., Li Q., Wu Y., Zhang H., Saiyin H., Liu X., Zhang X., Shi Q., Yu L. (2005). Aurora C is directly associated with Survivin and required for cytokinesis. Genes Cells.

[88] Bayliss R., Sardon T., Vernos I., Conti E. (2003). Structural basis of Aurora—A activation by TPX2 at the mitotic spindle. Mol. Cell.

[89] Eyers P.A., Maller J.L. (2004). Regulation of Xenopus Aurora a activation by TPX2. J. Biol. Chem..

[90] Xu J., Wu X., Zhou W.H., Liu A.W., Wu J.B., Deng J.Y., Yue C.F., Yang S.B., Wang J., Yuan Z.Y. (2013). Aurora-A identifies early recurrence and poor prognosis and promises a potential therapeutic target in triple negative breast cancer. PLoS One.

[91] Carmena M., Earnshaw W.C. (2003). The cellular geography of aurora kinases. Nat. Rev. Mol. Cell Biol..

[92] Moore A., Wordeman L. (2004). C-terminus of mitotic centromere-associated kinesin (MCAK) inhibits its lattice-stimulated ATPase activity. Biochem. J..

[93] Romanelli A., Clark A., Assayag F., Chateau-Joubert S., Poupon M.F., Servely J.L., Fontaine J.J., Liu X., Spooner E., Goodstal S. (2012). Inhibiting Aurora kinases reduces tumor growth and suppresses tumor recurrence after chemotherapy in patient-derived Triple-Negative Breast Cancer xenografts. Mol. Cancer Ther..

[94] Sebastian S., Settleman J., Reshkin S.J., Azzariti A., Bellizzi A., Paradiso A. (2006). The complexity of targeting EGFR signaling in cancer: From expression to turnover. Biochim. Biophys. Acta.

[95] Shiu K.K., Tan D.S., Reis-Filho J.S. (2008). Development of therapeutic approaches to “triple negative” phenotype breast cancer. Expert Opin. Ther. Targets.

[96] Corkery B., Crown J., Clynes M., O’Donovan N. (2009). Epidermal growth factor receptor as a potential therapeutic target in triple-negative breast cancer. Ann. Oncol..

[97] Duffy M.J., O’Donovan N., Crown J. (2011). Use of molecular markers for predicting therapy response in cancer patients. Cancer Treat. Rev..

[98] Hoadley K.A., Weigman V.J., Fan C., Sawyer L.R., He X., Troester M.A., Sartor C.I., Rieger-House T., Bernard P.S., Carey L.A. (2007). EGFR associated expression profiles vary with breast tumor subtype. BMC Genomics.

[99] O’Shaughnessy J., Weckstein D., Vukelja S. Preliminary results of a randomized phase II study of weekly irinotecan/carboplatin with or without cetuximab in patients with metastatic breast cancer. Proceedings of the SABCS.

[100] Carey L.A., Rugo H.S., Marcom P.K., Mayer E.L., Esteva F.J., Ma C.X., Liu M.C., Storniolo A.M., Rimawi M.F., Forero-Torres A. (2012). TBCRC 001: Randomized phase II study of cetuximab in combination with carboplatin in stage IV triple-negative breast cancer. J. Clin. Oncol..

[101] Masuda H., Zhang D., Bartholomeusz C., Doihara H., Hortobagyi G.N., Ueno N.T. (2012). Role of epidermal growth factor receptor in breast cancer. Breast Cancer Res. Treat..

[102] Gelmon K., Dent R., Mackey J.R., Laing K., McLeod D., Verma S. (2012). Targeting triple-negative breast cancer: Optimising therapeutic outcomes. Ann. Oncol..

[103] Agrawal A., Gutteridge E., Gee J.M., Nicholson R.I., Robertson J.F. (2005). Overview of tyrosine kinase inhibitors in clinical breast cancer. Endocr. Relat. Cancer.

[104] Twelves C., Trigo J.M., Jones R., de Rosa F., Rakhit A., Fettner S., Wright T., Baselga J. (2008). Erlotinib in combination with capecitabine and docetaxel in patients with metastatic breast cancer: A dose-escalation study. Eur. J. Cancer.

[105] Ciardiello F., Troiani T., Caputo F., de Laurentiis M., Tortora G., Palmieri G., de Vita F., Diadema M.R., Orditura M., Colantuoni G. (2006). Phase II study of gefitinib in combination with docetaxel as first-line therapy in metastatic breast cancer. Br. J. Cancer..

[106] Litzenburger B.C., Creighton C.J., Tsimelzon A., Chan B.T., Hilsenbeck S.G., Wang T., Carboni J.M., Gottardis M.M., Huang F., Chang J.C. (2011). High IGF-IR activity in triple-negative breast cancer cell lines and tumorgrafts correlates with sensitivity to anti-IGF-IR therapy. Clin. Cancer Res..

[107] Yang Y., Yee D. (2012). Targeting insulin and insulin-like growth factor signaling in breast cancer. J. Mammary Gland Biol. Neoplasia.

[108] Huang F., Greer A., Hurlburt W., Han X., Hafezi R., Wittenberg G.M., Reeves K., Chen J., Robinson D., Li A. (2009). The mechanisms of differential sensitivity to an insulin-like growth factor-1 receptor inhibitor (BMS-536924) and rationale for combining with EGFR/HER2 inhibitors. Cancer Res..

[109] Rensing K.L., Houttuijn Bloemendaal F.M., Weijers E.M., Richel D.J., Büller H.R., Koolwijk P., van der Loos C.M., Twickler T.B., von der Thüsen J.H. (2010). Could recombinant insulin compounds contribute to adenocarcinoma progression by stimulating local angiogenesis?. Diabetologia.

[110] Rozengurt E., Sinnett-Smith J., Kisfalvi K. (2010). Crosstalk between insulin/insulin-like growth factor-1 receptors and G protein-coupled receptor signaling systems: A novel target for the antidiabetic drug metformin in pancreatic cancer. Clin. Cancer Res..

[111] Shi Y., Yan H., Frost P., Gera J., Lichtenstein A. (2005). Mammalian target of rapamycin inhibitors activate the AKT kinase in multiple myeloma cells by up-regulating the insulin-like growth factor receptor/insulin receptor substrate-1/phosphatidylinositol 3-kinase cascade. Mol. Cancer Ther..

[112] O’Reilly K.E., Rojo F., She Q.B., Solit D., Mills G.B., Smith D., Lane H., Hofmann F., Hicklin D.J., Ludwig D.L. (2006). mTOR inhibition induces upstream receptor tyrosine kinase signaling and activates Akt. Cancer Res..

[113] Wan X., Harkavy B., Shen N., Grohar P., Helman L.J. (2007). Rapamycin induces feedback activation of Akt signaling through an IGF-1R-dependent mechanism. Oncogene.

[114] Clark A.S., West K., Streicher S., Dennis P.A. (2002). Constitutive and inducible Akt activity promotes resistance to chemotherapy, trastuzumab, or tamoxifen in breast cancer cells. Mol. Cancer Ther..

[115] Becker M.A., Ibrahim Y.H., Cui X., Lee A.V., Yee D. (2011). The IGF pathway regulates ER through a S6K1-dependent mechanism in breast cancer cells. Mol. Endocrinol..

[116] Fukazawa H., Noguchi K., Murakami Y., Uehara Y. (2002). Mitogen-activated protein/extracellular signal-regulated kinase kinase (MEK) inhibitors restore anoikis sensitivity in human breast cancer cell lines with a constitutively activated extracellular-regulated kinase (ERK) pathway. Mol. Cancer Ther..

[117] Brachmann S.M., Hofmann I., Schnell C., Fritsch C., Wee S., Lane H., Wang S., Garcia-Echeverria C., Maira S.M. (2009). Specific apoptosis induction by the dual PI3K/mTor inhibitor NVP-BEZ235 in HER2 amplified and PIK3CA mutant breast cancer cells. Proc. Natl. Acad. Sci. USA.

[118] Bertucci F., Finetti P., Cervera N., Charafe-Jauffret E., Mamessier E., Adélaïde J., Debono S., Houvenaeghel G., Maraninchi D., Viens P. (2006). Gene expression profiling shows medullary breast cancer is a subgroup of basal breast cancers. Cancer Res..

[119] Vinayak S., Gray R.J., Adams S., Jensen K.C., Manola J., Afghahi A., Goldstein L.J., Ford J.M., Badve S.S., Telli M.L. (2014). Association of increased tumor-infiltrating lymphocytes (TILs) with immunomodulatory (IM) triple-negative breast cancer (TNBC) subtype and response to neoadjuvant platinum-based therapy in PrECOG0105. J. Clin. Oncol..

[120] Ghiotto M., Gauthier L., Serriari N., Pastor S., Truneh A., Nunès J.A., Olive D. (2010). PD-L1 and PD-L2 differ in their molecular mechanisms of interaction with PD-1. Int. Immunol..

[121] Muenst S., Schaerli A.R., Gao F., Däster S., Trella E., Droeser R.A., Muraro M.G., Zajac P., Zanetti R., Gillanders W.E. (2014). Expression of programmed death ligand 1 (PD-L1) is associated with poor prognosis in human breast cancer. Breast Cancer Res. Treat..

[122] Basu G.D., Ghazalpour A., Gatalica Z., Anderson K.S., McCullough A.E., Spetzer D.B., Pockaj B.A. (2014). Expression of novel immunotherapeutic targets in triple-negative breast cancer. J. Clin. Oncol..

[123] Gibson G.R., Qian D., Ku J.K., Lai L.L. (2005). Metaplastic breast cancer: Clinical features and outcomes. Am. Surg..

[124] Hennessy B.T., Gonzalez-Angulo A.M., Stemke-Hale K., Gilcrease M.Z., Krishnamurthy S., Lee J.S., Fridlyand J., Sahin A., Agarwal R., Joy C. (2009). Characterization of a naturally occurring breast cancer subset enriched in epithelial-to-mesenchymal transition and stem cell characteristics. Cancer Res..

[125] Shin S.Y., Rath O., Zebisch A., Choo S.M., Kolch W., Cho K.H. (2010). Functional roles of multiple feedback loops in extracellular signal-regulated kinase and Wnt signaling pathways that regulate epithelial-mesenchymal transition. Cancer Res..

[126] Hayes M.J., Thomas D., Emmons A., Giordano T.J., Kleer C.G. (2008). Genetic changes of Wnt pathway genes are common events in metaplastic carcinomas of the breast. Clin. Cancer Res..

[127] Hao J., Ao A., Zhou L., Murphy C.K., Frist A.Y., Keel J.J., Thorne C.A., Kim K., Lee E., Hong C.C. (2013). Selective small molecule targeting β-catenin function discovered by *in vivo* chemical genetic screen. Cell Rep..

[128] MacDonald B.T., Tamai K., He X. (2009). Wnt/beta-catenin signaling: Components, mechanisms, and diseases. Dev. Cell.

[129] Verbeek B.S., Vroom T.M., Adriaansen-Slot S.S., Ottenhoff-Kalff A.E., Geertzema J.G., Hennipman A., Rijksen G. (1996). c-Src protein expression is increased in human breast cancer. An immunohistochemical and biochemical analysis. J. Pathol..

[130] Tryfonopoulos D., Walsh S., Collins D.M., Flanagan L., Quinn C., Corkery B., McDermott E.W., Evoy D., Pierce A., O’Donovan N. (2011). Src: A potential target for the treatment of triple-negative breast cancer. Ann. Oncol..

[131] Talpaz M., Shah N.P., Kantarjian H., Donato N., Nicoll J., Paquette R., Cortes J., O’Brien S., Nicaise C., Bleickardt E. (2006). Dasatinib in imatinib-resistant Philadelphia chromosome-positive leukemias. N. Engl. J. Med..

[132] Finn R.S., Dering J., Ginther C., Wilson C.A., Glaspy P., Tchekmedyian N., Slamon D.J. (2007). Dasatinib, an orally active small molecule inhibitor of both the src and abl kinases, selectively inhibits growth of basal-type/“triple-negative” breast cancer cell lines growing *in vitro*. Breast Cancer Res. Treat..

[133] Huang F., Reeves K., Han X., Fairchild C., Platero S., Wong T.W., Lee F., Shaw P., Clark E. (2007). Identification of candidate molecular markers predicting sensitivity in solid tumors to dasatinib: Rationale for patient selection. Cancer Res..

[134] Finn R.S., Bengala C., Ibrahim N., Roché H., Sparano J., Strauss L.C., Fairchild J., Sy O., Goldstein L.J. (2011). Dasatinib as a single agent in triple-negative breast cancer: Results of an open-label phase 2 study. Clin. Cancer Res..

[135] Kim E.M., Mueller K., Gartner E., Boerner J. (2013). Dasatinib is synergistic with cetuximab and cisplatin in triple-negative breast cancer cells. J. Surg. Res..

[136] Mezi S., Todi L., Orsi E., Angeloni A., Mancini P. (2012). Involvement of the Src-cortactin pathway in migration induced by IGF-1 and EGF in human breast cancer cells. Int. J. Oncol..

[137] Fornier M.N., Morris P.G., Abbruzzi A., D’Andrea G., Gilewski T., Bromberg J., Dang C., Dickler M., Modi S., Seidman A.D. (2011). A phase I study of dasatinib and weekly paclitaxel for metastatic breast cancer. Ann. Oncol..

[138] Somlo G., Atzori F., Strauss L.C., Geese W.J., Specht J.M., Gradishar W.J., Rybicki A., Sy O., Vahdat L.T., Cortes J. (2013). Dasatinib plus capecitabine for advanced breast cancer: Safety and efficacy in phase I study CA180004. Clin. Cancer Res..

[139] Ueno N.T., Zhang D. (2011). Targeting EGFR in triple negative breast cancer. J. Cancer.

[140] Rydén L., Ferno M., Stal O., Linderholm B., Ostman A., Jirstrom K. (2009). Vascular endothelial growth factor receptor 2 is a significant negative prognostic biomarker in triple-negative breast cancer: Results from a controlled randomised trial of premenopausal breast cancer. Cancer Res..

[141] Jayson G.C., Haas S.D., Delmar P., Miles D.W., Shah M.A., van Cutsem E., Carmeliet P., Hegde P., Wild N., Scherer S.J. Evaluation of plasma VEGFA as a potential predictive pan-tumour biomarker for bevacizumab. Presented at the European Multidisciplinary Cancer Congress.

[142] Yang S.X., Steinberg S.M., Nguyen D., Wu T.D., Modrusan Z., Swain S.M. (2008). Gene expression profile and angiogenic marker correlates with response to neoadjuvant bevacizumab followed by bevacizumab plus chemotherapy in breast cancer. Clin. Cancer Res..

[143] Linderholm B.K., Hellborg H., Johansson U., Elmberger G., Skoog L., Lehtiö J., Lewensohn R. (2009). Significantly higher levels of vascular endothelial growth factor (VEGF) and shorter survival times for patients with primary operable triple-negative breast cancer. Ann. Oncol..

[144] Lindholm E.M., Kristian A., Nalwoga H., Krüger K., Nygård S., Akslen L.A., Mælandsmo G.M., Engebraaten O. (2012). Effect of antiangiogenic therapy on tumor growth, vasculature and kinase activity in basal- and luminal-like breast cancer xenografts. Mol. Oncol..

[145] Miller K., Wang M., Gralow J., Dickler M., Cobleigh M., Perez E.A., Shenkier T., Cella D., Davidson N.E. (2007). Paclitaxel plus bevacizumab *versus* paclitaxel alone for metastatic breast cancer. N. Engl. J. Med..

[146] Miles D.W., Chan A., Dirix L.Y., Cortés J., Pivot X., Tomczak P., Delozier T., Sohn J.H., Provencher L., Puglisi F. (2010). Phase III study of bevacizumab plus docetaxel compared with placebo plus docetaxel for the first-line treatment of human epidermal growth factor receptor 2-negative metastatic breast cancer. J. Clin. Oncol..

[147] Robert N.J., Diéras V., Glaspy J., Brufsky A.M., Bondarenko I., Lipatov O.N., Perez E.A., Yardley D.A., Chan S.Y., Zhou X. (2011). RIBBON-1: Randomized, double-blind, placebo-controlled, phase III trial of chemotherapy with or without bevacizumab for first-line treatment of human epidermal growth factor receptor 2-negative, locally recurrent or metastatic breast cancer. J. Clin. Oncol..

[148] Miles D.W., Diéras V., Cortés J., Duenne A.A., Yi J., O’Shaughnessy J. (2013). First-line bevacizumab in combination with chemotherapy for HER2-negative metastatic breast cancer: Pooled and subgroup analyses of data from 2447 patients. Ann. Oncol..

[149] Rossari J.R., Metzger-Filho O., Paesmans M., Saini K.S., Gennari A., de Azambuja E., Piccart-Gebhart M. (2012). Bevacizumab and breast cancer: A meta-analysis of first-line phase III studies and a critical reappraisal of available evidence. J. Oncol..

[150] Cancer Genome Atlas Network (2012). Comprehensive molecular portraits of human breast tumours. Nature.

[151] Stemke-Hale K., Gonzalez-Angulo A.M., Lluch A., Neve R.M., Kuo W.L., Davies M., Carey M., Hu Z., Guan Y., Sahin A. (2008). An integrative genomic and proteomic analysis of PIK3CA, PTEN, and AKT mutations in breast cancer. Cancer Res..

[152] Sohn J., Do K.A., Liu S., Chen H., Mills G.B., Hortobagyi G.N., Meric-Bernstam F., Gonzalez-Angulo A.M. (2013). Functional proteomics characterization of residual triple-negative breast cancer after standard neoadjuvant chemotherapy. Ann. Oncol..

[153] Baselga J., Semiglazov V., van Dam P., Manikhas A., Bellet M., Mayordomo J., Campone M., Kubista E., Greil R., Bianchi G. (2009). Phase II randomized study of neoadjuvant everolimus plus letrozole compared with placebo plus letrozole in patients with estrogen receptor-positive breast cancer. J. Clin. Oncol..

[154] Jerusalem G., Fasolo A., Dieras V., Cardoso F., Bergh J., Vittori L., Zhang Y., Massacesi C., Sahmoud T., Gianni L. (2011). Phase I trial of oral mTOR inhibitor everolimus in combination with trastuzumab and vinorelbine in pre-treated patients with HER2-overexpressing metastatic breast cancer. Breast Cancer Res. Treat..

[155] Yunokawa M., Koizumi F., Kitamura Y., Katanasaka Y., Okamoto N., Kodaira M., Yonemori K., Shimizu C., Ando M., Masutomi K. (2012). Efficacy of everolimus, a novel mTOR inhibitor, against basal-like triple-negative breast cancer cells. Cancer Sci..

[156] Serra V., Markman B., Scaltriti M., Eichhorn P.J., Valero V., Guzman M., Botero M.L., Llonch E., Atzori F., di Cosimo S. (2008). NVP-BEZ235, a dual PI3K/mTOR inhibitor, prevents PI3K signaling and inhibits the growth of cancer cells with activating PI3K mutations. Cancer Res..

[157] Montero J.C., Esparís-Ogando A., Re-Louhau M.F., Seoane S., Abad M., Calero R., Ocana A., Pandiella A. (2014). Active kinase profiling, genetic and pharmacological data define mTOR as an important common target in triple-negative breast cancer. Oncogene.

[158] Juvekar A., Burga L.N., Hu H., Lunsford E.P., Ibrahim Y.H., Balmañà J., Rajendran A., Papa A., Spencer K., Lyssiotis C.A. (2012). Combining a PI3K inhibitor with a PARP inhibitor provides an effective therapy for BRCA1-related breast cancer. Cancer Discov..

[159] Doane A.S., Danso M., Lal P., Donaton M., Zhang L., Hudis C., Gerald W.L. (2006). An estrogen receptor-negative breast cancer subset characterized by a hormonally regulated transcriptional program and response to androgen. Oncogene.

[160] Farmer P., Bonnefoi H., Becette V., Tubiana-Hulin M., Fumoleau P., Larsimont D., Macgrogan G., Bergh J., Cameron D., Goldstein D. (2005). Identification of molecular apocrine breast tumours by microarray analysis. Oncogene.

[161] Rouzier R., Perou C.M., Symmans W.F., Ibrahim N., Cristofanilli M., Anderson K., Hess K.R., Stec J., Ayers M., Wagner P. (2005). Breast cancer molecular subtypes respond differently to preoperative chemotherapy. Clin. Cancer Res..

[162] Parker J.S., Mullins M., Cheang M.C., Leung S., Voduc D., Vickery T., Davies S., Fauron C., He X., Hu Z. (2009). Supervised risk predictor of breast cancer based on intrinsic subtypes. J. Clin. Oncol..

[163] Fizazi K., Scher H.I., Molina A., Logothetis C.J., Chi K.N., Jones R.J., Staffurth J.N., North S., Vogelzang N.J., Saad F. (2012). Abiraterone acetate for treatment of metastatic castration-resistant prostate cancer: Final overall survival analysis of the COU-AA-301 randomised, double-blind, placebo-controlled phase 3 study. Lancet Oncol..

[164] Ryan C.J., Smith M.R., de Bono J.S., Molina A., Logothetis C.J., de Souza P., Fizazi K., Mainwaring P., Piulats J.M., Ng S. (2013). Abiraterone in metastatic prostate cancer without previous chemotherapy. N. Engl. J. Med..

[165] Nadal R., Taplin M.E., Bellmunt J. (2014). Enzalutamide for the treatment of prostate cancer: Results and implications of the AFFIRM trial. Future Oncol..

[166] Beer T.M., Armstrong A.J., Sternberg C.N., Higano C.S., Iversen P., Loriot Y., Rathkopf D.E., Bhattacharya S., Carles J., de Bono J.S. (2014). Enzalutamide in men with chemotherapy-naive metastatic prostate cancer (mCRPC): Results of phase III PREVAIL study. J. Clin. Oncol..

[167] Tannock I.F., de Wit R., Berry W.R., Horti J., Pluzanska A., Chi K.N., Oudard S., Théodore C., James N.D., Turesson I. (2004). Docetaxel plus prednisone or mitoxantrone plus prednisone for advanced prostate cancer. N. Engl. J. Med..

[168] Petrylak D.P., Tangen C.M., Hussain M.H., Lara P.N., Jones J.A., Taplin M.E., Burch P.A., Berry D., Moinpour C., Kohli M. (2004). Docetaxel and estramustine compared with mitoxantrone and prednisone for advanced refractory prostate cancer. N. Engl. J. Med..

[169] De Bono J.S., Oudard S., Ozguroglu M., Hansen S., Machiels J.P., Kocak I., Gravis G., Bodrogi I., Mackenzie M.J., Shen L. (2010). Prednisone plus cabazitaxel or mitoxantrone for metastatic castration-resistant prostate cancer progressing after docetaxel treatment: A randomised open-label trial. Lancet.

[170] Solit D.B., Zheng F.F., Drobnjak M., Münster P.N., Higgins B., Verbel D., Heller G., Tong W., Cordon-Cardo C., Agus D.B. (2002). 17-Allylamino-17-demethoxygeldanamycin induces the degradation of androgen receptor and HER2/neu and inhibits the growth of prostate cancer xenografts. Clin. Cancer Res..

[171] Bukau B., Weissman J., Horwich A. (2006). Molecular chaperones and protein quality control. Cell.

[172] Calderwood S.K. (2010). Heat shock proteins in breast cancer progression—A suitable case for treatment?. Int. J. Hyperth..

[173] Beliakoff J., Whitesell L. (2004). Hsp90: An emerging target for breast cancer therapy. Anticancer Drugs.

[174] Powers M.V., Workman P. (2006). Targeting of multiple signalling pathways by heat shock protein 90 molecular chaperone inhibitors. Endocr. Relat. Cancer.

[175] Zagouri F., Bournakis E., Koutsoukos K., Papadimitriou C.A. (2012). Heat shock protein 90 (hsp90) expression and breast cancer. Pharmaceuticals (Basel).

[176] Sequist L., Janne P., Sweney J., Walker J., Grayzel D., Lynch T. Phase 1/2 trial of the novel Hsp90 inhibitor, IPI-504, in patients with relapsed and/or refractory stage IIIb or stage IV non-small cell lung cancer (NSCLC) stratified by EGFR mutation status. Proceedings of the International Conference on Molecular Targets and Cancer Therapeutics.

[177] Bryson J., Infante J., Ramanathan R.K., Jones S.F., von Hoff D.D., Burris H.A. (2008). A phase I dose-escalation study of the safety and pharmacokinetics (PK) of the oral Hsp90 inhibitor SNX-5422. J. Clin. Oncol..

[178] Wagner A., Morgan J., Chugh R., Rosen L.S., George S., Gordon M.S., Devine C.M., van den Abbeele A.D., Grayzel D., Demetri G.D. (2008). Inhibition of heat shock protein 90 (Hsp90) with the novel agent IPI-504 in metastatic GIST following failure of tyrosine kinase inhibitors (TKIs) or other sarcomas: Clinical results from a phase I trial. J. Clin. Oncol..

[179] Elfiky A., Saif M.W., Beeram M., O’Brien S., Lammanna N., Castro J.E., Woodworth J., Perea R., Storgard C., von Hoff D.D. (2008). BIIB021, an oral, synthetic non-ansamycin Hsp90 inhibitor: Phase I experience. J. Clin. Oncol..

[180] Ide S., Motwani M., Jensen M.R., Wang J., Huseinovic N., Stiegler P., Wang X., Quadt C. (2009). Pharmacodynamics and pharmacokinetics of AUY922 in a phase I study of solid tumor patients. J. Clin. Oncol..

[181] Ramanathan R.K., Egorin M.J., Eiseman J.L., Ramalingam S., Friedland D., Agarwala S.S., Ivy S.P., Potter D.M., Chatta G., Zuhowski E.G. (2007). Phase I and pharmacodynamic study of 17-(allylamino)-17-demethoxygeldanamycin in adult patients with refractory advanced cancers. Clin. Cancer Res..

[182] Caldas-Lopes E., Cerchietti L., Ahn J.H., Clement C.C., Robles A.I., Rodina A., Moulick K., Taldone T., Gozman A., Guo Y. (2009). Hsp90 inhibitor PU-H71, a multimodal inhibitor of malignancy, induces complete responses in triple-negative breast cancer models. Proc. Natl. Acad. Sci. USA.

[183] Proia D.A., Zhang C., Sequeira M., Jimenez J.P., He S., Spector N., Shapiro G.I., Tolaney S., Nagai M., Acquaviva J. (2014). Preclinical activity profile and therapeutic efficacy of the HSP90 inhibitor ganetespib in triple-negative breast cancer. Clin. Cancer Res..

[184] Ying W., Du Z., Sun L., Foley K.P., Proia D.A., Blackman R.K., Zhou D., Inoue T., Tatsuta N., Sang J. (2012). Ganetespib, a unique triazolone-containing Hsp90 inhibitor, exhibits potent antitumor activity and a superior safety profile for cancer therapy. Mol. Cancer Ther..

[185] Liu X., Gomez-Pinillos A., Ferrari A.C. (2010). Simultaneous targeting of the androgen receptor and PI3K/mTOR pathway in androgen-dependent and androgen-independent prostate cancer cells. J. Clin. Oncol..

